# A Qualitative Model of the Differentiation Network in Chondrocyte Maturation: A Holistic View of Chondrocyte Hypertrophy

**DOI:** 10.1371/journal.pone.0162052

**Published:** 2016-08-31

**Authors:** Johan Kerkhofs, Jeroen Leijten, Johanna Bolander, Frank P. Luyten, Janine N. Post, Liesbet Geris

**Affiliations:** 1 Biomechanics Research Unit, University of Liège, Liège, Belgium; 2 Biomechanics section, KU Leuven, Leuven, Belgium; 3 Prometheus, the Leuven R&D division of skeletal tissue engineering, KU Leuven, Leuven, Belgium; 4 Skeletal Biology and Engineering Research Center, KU Leuven, Leuven, Belgium; 5 Developmental BioEngineering, MIRA Institute for biomedical technology and technical medicine, University of Twente, Enschede, The Netherlands; University of Southampton, UNITED KINGDOM

## Abstract

Differentiation of chondrocytes towards hypertrophy is a natural process whose control is essential in endochondral bone formation. It is additionally thought to play a role in several pathophysiological processes, with osteoarthritis being a prominent example. We perform a dynamic analysis of a qualitative mathematical model of the regulatory network that directs this phenotypic switch to investigate the influence of the individual factors holistically. To estimate the stability of a SOX9 positive state (associated with resting/proliferation chondrocytes) versus a RUNX2 positive one (associated with hypertrophy) we employ two measures. The robustness of the state in canalisation (size of the attractor basin) is assessed by a Monte Carlo analysis and the sensitivity to perturbations is assessed by a perturbational analysis of the attractor. Through qualitative predictions, these measures allow for an *in silico* screening of the effect of the modelled factors on chondrocyte maintenance and hypertrophy. We show how discrepancies between experimental data and the model’s results can be resolved by evaluating the dynamic plausibility of alternative network topologies. The findings are further supported by a literature study of proposed therapeutic targets in the case of osteoarthritis.

## Introduction

### Relevance of developmental biology to bone tissue engineering

In bone tissue engineering (TE) strategies, progenitor cells are combined with a bioartificial scaffold and/or specific growth factors to initiate a process of new bone formation with the aim of addressing an unmet clinical need in treating large bone defects [1]. For TE constructs, an important obstacle for clinical translation lies in controlling the variability in the cell populations available for this approach. Typically these populations are heterogeneous and may furthermore differ dramatically in behaviour in different individuals [2]. This may partly explain why the tissue engineering approach currently lacks the reproducibility essential for successful clinical translation. In line with the recently introduced paradigm of `developmental engineering', a more fundamental understanding of the biological processes involved in bone formation and repair can therefore be of great use in improving the efficacy of these constructs [3]. Given that the bone defect healing process is in effect a reiteration of developmental bone formation, albeit in a different context and microenvironment, we can use the wealth of studies on developmental biology that is available to provide biological data [4,5].

### Chondrocyte hypertrophy

Specifically, we model the activity and interaction of the set of genes that is thought to be crucial for the late differentiation of chondrocytes in the growth plate, nature’s engine for bone growth [6]. This process is called hypertrophy, a crucial step in endochondral ossification, the most common bone forming process responsible for the formation of the appendicular and axial skeleton [7]. During hypertrophic differentiation, the growth plate chondrocytes undergo a differentiation cascade that takes them from round resting zone chondrocytes through proliferating chondrocytes in columnar organisation to enlarged hypertrophic chondrocytes. Hypertrophic chondrocytes secrete catabolic factors to degrade the cartilage matrix and they attract blood vessels and accompanying osteoblast precursors to invade and form the bone primordia. These characteristics make hypertrophic chondrocytes well suited for incorporation into bone TE constructs [8,9]. These same characteristics also separate them distinctively from permanent cartilage, such as articular cartilage, which—under healthy conditions—is not susceptible to hypertrophic differentiation [10–12]. Yet, ectopic hypertrophy will occur under pathophysiological conditions such as in osteoarthritis (OA). This phenotypic switch to hypertrophy can furthermore drive other pathophysiological processes such as heterotopic ossification and intervertebral disc calcification [13–15]. Genetic studies imply that faults in structural proteins do not appear to be decisive in developing OA, leading to the interpretation that the aetiology is regulatory rather than structural [16]. Given that *RUNX2* heterozygote knockout mice show resistance to OA development in conjunction with decreased *MMP13* expression, it is indeed likely that chondrocyte hypertrophy will play at the least a contributory role in OA pathophysiology [17,18].

### Mathematical modelling of regulatory network overseeing hypertrophy

A curse in cartilage TE and a boon for bone TE, understanding the cellular machinery underlying hypertrophy is equally essential to both endeavours. For this reason, along with its occurrence in several pathophysiological processes, this study focuses on the hypertrophic fate decision in chondrogenic differentiation. We hence aim to increase insight into the molecular networks underpinning the prevention, induction or propagation of chondrocyte hypertrophy by incorporating biological information into a qualitative mathematical model. Depending on the quantity and the form of information that is available, many modelling formalisms have been introduced suited to perform dynamical studies of networks [19–21]. Many formalisms can be categorized as discrete or continuous, deterministic or stochastic, qualitative or quantitative, numerical or analytical, but hybrids falling into neither category are abound [22–27]. Due to the difficulty in establishing a suitable *in vitro* model and the technological obstacles in obtaining *in vivo* data in cartilage biology, detailed kinetic data are scarce, necessitating a qualitative approach. Here we employ a qualitative approach with a limited time resolution [28]. In short, this framework allows an investigation of the qualitative response to different doses of growth factors taking into account a simplified dynamics where biological interactions occur on two disparate time scales.

### Objectives

Specifically, in this work we attempt to deduce the contributions of individual factors to the stability of the chondrocyte phenotype and to hypertrophic differentiation. To this end, we constructed an elaborate network incorporating many factors deemed vital to this process. The gene network centres on the primordial transcription factors SOX9 and RUNX2 whose presence or absence is decisive for cell fate decisions. Indeed, persistent *SOX9* expression is required for permanent cartilage while overexpression of *RUNX2* induces hypertrophy and is an important earmark of hypertrophy in transient cartilage [29,30]. In combining several signalling pathways we are able to establish a more holistic view of cartilage biology, and the extent to which different factors impact on fate decisions as their effects ripple through this regulatory network.

## Materials & Methods

**Fig 1:**
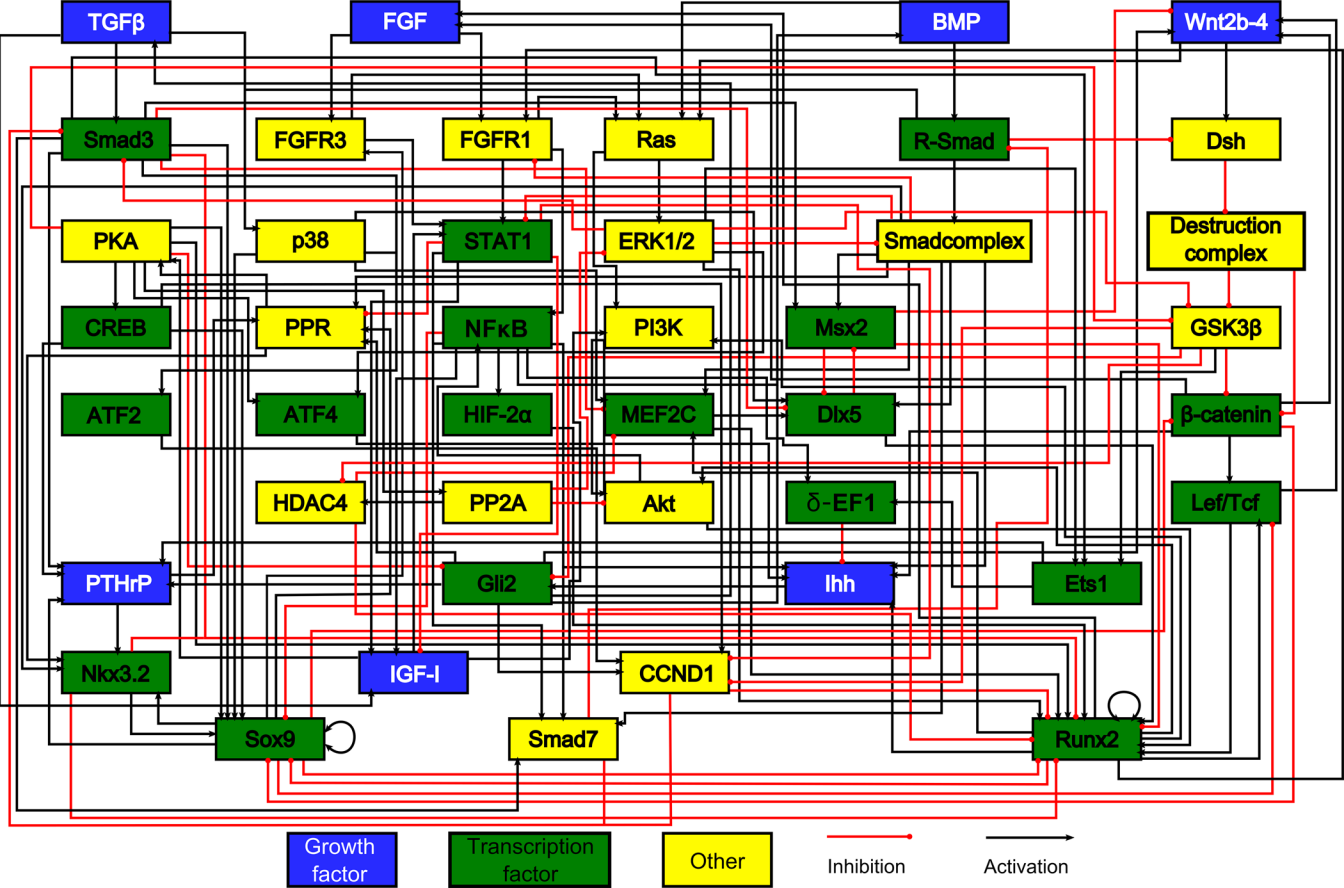
The model’s chondrocyte gene network. Every box represents a gene, its protein or in some cases a complex of them. The interactions are represented by red and black lines if they are inhibitory and stimulatory, respectively. Blue boxes denote growth factors, green boxes are transcription factors, yellow boxes do not belong to either category. Reproduced from [28].

The network analysed here is intended to simulate the phenotypic switch from proliferation to hypertrophy in growth plate chondrocytes. The model is based on an extensive literature study and is an expansion of a previously published model [31]. Uniquely, this novel model is focused on the control of crucial genes, SOX9 and RUNX2. In particular, Insulin-like growth factor (IGF) signalling and the phosphatidylinositol-3-kinase (PI3K)–AKT pathway are among the pathways that were added in light of their importance in chondrogenesis and association with SOX9 and RUNX2 control [32]. Moreover, transcription factors that contribute to the transition from proliferation to hypertrophy have been included. Of note, we incorporated hypoxia-inducible factor-2α (HIF-2α), which is hypothesized to be a causative factor in ectopic hypertrophy in the onset of osteoarthritis [33,34]; activating transcription factor 4 (ATF4), the absence of which accelerates hypertrophy in mouse models [35], and δ-EF1, an inhibitor of IHH expression [36]. Additionally, we attempted to improve our prediction of parathyroid hormone-related protein (PTHRP) and Indian hedgehog (IHH) expression, two major determinants of growth plate biology, by adding more upstream interactions [6]. Jointly these changes allow an improved simulation of the factors that play a role in the hypertrophic switch. It is of note that the network is an abridged version in the sense that only biological factors that receive multiple inputs are included whereas factors that simply act as a relay are omitted. For example in the linear pathway sequence WNT to Frizzled (FZ) to Dishevelled (DSH), FZ is not included in the model since no interactions of other pathways impede on these receptors in our model. Fig 1 shows the network. Sources for novel and old interactions are given in S1 File.

As seen in Fig 1, both SOX9 and RUNX2 are extensively regulated. Here we briefly discuss the mechanisms that were included in this work. Several transcription factors were found to act upstream of RUNX2 in chondrogenic differentiation. The canonical Wnt effector, β-CATENIN, was shown to induce *Runx2* expression in association with LEF/TCF [37]. DLX5, downstream of BMP signaling, HIF-2α and MEF2C also positively regulate *Runx2* promoter activity [33,38–40]. Once induced, RUNX2 can increase its own expression through autoregulation. These stimuli are kept in check through transcriptional repression by MSX2 and an NKX3.2-SMAD complex [41,42]. The activity of the translated RUNX2 protein is additionally modified by a collection of pathways. Several factors have been shown to increase RUNX2 activity. A first implicated pathway is PI3K-AKT signaling, which increases the DNA binding ability of RUNX2 [43]. Another stimulus is provided by phosphorylation of RUNX2 by ERK signaling, resulting in higher activity [44]. PKA signaling can likewise phosphorylate RUNX2 and increase expression of target genes [45]. RUNX2 activity can furthermore be increased through an interaction with DLX5 [46]. Other factors decrease RUNX2 activity. SOX9 can bind directly to RUNX2 and thereby impairs its function [47]. Similarly, MSX2 binds to RUNX2 to the detriment of its transcriptional ability [48]. SMAD3 and HDAC4 associate to prevent transcription by RUNX2[49]. In a last inhibitory mechanism, CCND1 can phosphorylate RUNX2 resulting in proteasome-dependent degradation [50].

*Sox9* expression is promoted by CREB (downstream of PKA) and through a P38-dependent mechanism [51,52]. A positive feedback loop through NKX3.2 and autoregulation can then help sustain expression [53–55]. Posttranslational mechanisms additionally modify SOX9 activity. In particular, SOX9 activity is greatly increased after phosphorylation by PKA [56]. In response to TGFβ signalling, SMAD3 helps recruit transcriptional co-activators to the SOX9 transcriptional complex [57] Finally, binding of SOX9 with RUNX2 and β-CATENIN results in mutual inhibition [58,59].

To study the dynamical behaviour of this network we use a qualitative framework, which is described in detail in Kerkhofs et al. [28]. In this framework, each node is associated with a continuous value between 0, indicating no activity, and 1, the maximal activity. This value is determined dynamically by additive combination of the activities of upstream nodes. To tune the activities of the network, a saturation factor (which is the same for all nodes) is included which determines how much of the positive signals must be present for a node to be fully active. The influence of this factor is discussed in S2 File. The system is updated in a discrete fashion by general asynchronous updating with two priority classes, as introduced by Fauré et al [60]. The priority classes divide the interactions into a fast and a slow class, each controlling one variable per node. Activity is determined by multiplying a slow variable, which incorporates interactions that involve transcription or mRNA degradation etc, and a fast variable, modelling the influence of interactions involving post-translational modifications or inhibition through binding etc. Both variables are limited to the interval [0,1]. For example, the PTH/PTHrP receptor (PPR) activity is a result of both the slow variable, indicating transcription by SOX9, GLI or SMAD complex, and the fast variable, indicating the presence of PTHRP to activate the transcribed receptor. Slow variables are only updated when all fast variables are in equilibrium, meaning that the fast variable of PPR will always be updated before the slow one (or any other slow variable). The equations used can be found in S3 File. When all variables are in equilibrium, the system has reached a steady state. We are primarily interested in these steady states or attractors since they dominate the long term behaviour of the network.

The attractors of this network will be linked with biologically meaningful states by interpreting the expression of selected genes in much the same way as marker genes are used in biological models to define cell state. In practice, any attractor is categorized as either a proliferating (stable) chondrocyte with SOX9 activity or as hypertrophic in the event of RUNX2 activity. In case neither shows activity we categorize the state as ‘None’. We cannot necessarily connect a biological fate to this outcome, as depending on the circumstances this might plausibly result for example in apoptosis or the adoption of an adipogenic phenotype. In any case, the ‘None’ state indicates that neither a chondrogenic nor a hypertrophic fate is predicted.

We use two measures to assess the phenotypical stability of certain biologically relevant stable states. A first measure is the size of the attractor basin [61]. Due to the exponential growth of the state space with the size of the network, it is not feasible to investigate a significant fraction of the state space in larger networks. Moreover, the inherent stochasticity of the updating method precludes a definitive assignment of any one point in state space to one specific attractor. As a proxy, we use a Monte Carlo method to assess the likelihood of reaching certain attractors from random initial conditions. In particular, we initialise the system in a random initial state and determine to which stable state it converges. By repeating this process numerous times we can estimate the likelihood of an initial state ending up in a particular attractor. This likelihood then constitutes a measure of the phenotypical stability of the attractor, similar to Waddington’s concept of canalisation. This is a way to gauge the size of the attractor basins, the region of state space that can flow to a certain attractor, and by extension the stability (to perturbation in initial conditions) of the respective stable states.

To examine the importance of a certain node in influencing the outcome of the simulation, we have performed an *in silico* screening where all nodes in the network were individually perturbed. We simulate two cases: the first case simulates the event of a knockout (loss of function), where the node is effectively removed from the network by holding its activity to zero at all times. The second case is the opposite of the first one, i.e. the overactivation (gain of function) of the node. Here the activity is kept at a maximum throughout the simulation. For each node these simulations were done three times for 10.000 instances providing an estimate of the attractor basin size for each of the cases. Since we are interested in the relative contribution of each node to the attractor basin size, as a proxy for the phenotypical stability, we compare the results to the baseline value of the wild type network.

This first measure tallies the attractors that random initial states end up in to assert how likely it is to reach certain states. However, one can argue that many of these states would have a negligible prevalence in the relatively stable and tightly orchestrated processes of embryonic development and mature tissue homeostasis. In consequence, the majority of initial states might not have a biological counterpart, but could dominate the final outcome and obscure results for more relevant states.

The second measure remedies this potential bias by examining the states with the highest biological relevance, the stable states. We chose to investigate the effect of a large perturbation on these states, which are the computational equivalents of both transgenic and knockout animals and cellular *in vitro* knockdown and overexpression models. For each of the attractors we maximised and/or minimised the activity of each node in turn. For nodes that were already at a maximal or minimal value only one perturbation was possible. The perturbation was applied for a certain amount of time (updates) after which the system was allowed to settle. We set the perturbation time up to the point that a further increase had no effect on the result. We performed 100 simulations for each perturbation and tracked the outcome to estimate the probability of transiting to each of the individual states. By evaluating different perturbation sizes one can also employ this method to find out which nodes require the smallest perturbation to leave a particular steady state. This was done for the transitions of the SOX9 to ‘None’ states as well as the SOX9 to RUNX2 state.

By averaging the transition probabilities for all nodes of a particular stable state we can determine the transition probabilities for that stable state as a whole to a random perturbation. Where both overactivation and inactivation of a node were possible, the result of this node was taken as the average of these two cases. These transition possibilities between the stable states of the network determine the transition matrix. We can regard this system and consequently the process of differentiation as a Markov chain consisting of the three stable states [62,63]. In the sense of a Markov chain, this system has one stationary distribution regardless of its initial condition which follows from the transition matrix. From the viewpoint of a single cell, the probability of a state in the stationary distribution is proportional to the time a cell will spend in this state when continuously affected by discrete random perturbations. Equivalently, from a population view the probability of a state is the fraction of cells in a continuously perturbed population that show the phenotype attributed to this specific state.

To empirically validate our predictions, we activated distinct signalling pathways and analysed the gene expression levels of the stimulated cells. The ethical committee for Human Medical Research (KU Leuven, ML7861) approved all procedures. Periost was obtained from the patient after signing an informed consent. All patients underwent a surgical intervention for a lengthening or correction of the tibia and at that time point the biopsies were taken, and only used for scientific research. In specific, we seeded human periosteal derived stem cells at 10.000 cells per cm^2^. To minimize unpredictable serum effects, we starved the cells in culture medium containing 0.1% BSA for 16 hours post-attachment and subsequently exposed the cells to the signalling pathway activating growth factors using a serum free chemically defined medium as previously described [64] with the removal of 3,3',5-triiodo-L-thyronine. To map the cellular effects WNT and BMP signaling activation, cells were stimulated with vehicle, 100 ng/ml of BMP2 (Medtronic, Minnesota, U.S) and/or 100 ng/ml of WNT3A (R&D Systems, Oxon, U.K). To also mimic the different cellular ground states that we explore in our model, we included the following experiment. These cells were then washed and received both BMP2 and WNT3A. After 24 hours of stimulation, cells were lysed. The mRNA was isolated using an RNeasy mini kit (Qiagen, Venlo, NL) and measured using a Nanodrop ND2000 (Thermo Scientific, Erembodegem, BE). Complementary DNA (cDNA) was synthesized using the RevertAid H Minus First Strand cDNA Synthesis Kit (Thermo Scientific, Erembodegem, BE) and 500 ng of nonamplified total RNA. For each condition a total of 20 ng of cDNA was amplified using a Fast Sybr green master mix (Applied Biosciences) and a Corbett rotor gene QPCR (Qiagen, Venlo, NL). All steps were performed according their respective manufacturer’s instructions. Gene expression was normalized on beta-actin (*ACTB*) expression levels, which was found to be stable reference gene. Primer sequences can be found in S1 Table. Gene expression results were analyzed by t-tests using R 3.1.1. [65].

## Results

The attractors, or stable states, of the network were identified numerically. They are given in Table 1. We found that the wild type network has 3 individual attractors, one SOX9 positive state, one RUNX2 positive state and a state where all the nodes are inactive, with the exception of some constitutively active nodes (the ‘None’ state). It is possible that some attractors with a very small attractor basin and that consequently have a very small likelihood of being reached were not detected in this analysis. However, any attractors with such a small basin of attraction would likely be biologically irrelevant.

**Table 1:** The stable states of the network. The activity of each node is given for each of the three attractors (SOX9, RUNX2 and None).

Node	SOX9	RUNX2	None
**WNT**	0	1	0
**DSH**	0	0,67	0
**IGF-I**	1	0	0
**R-SMAD**	0,19	0,67	0
**IHH**	0,4	0,61	0
**GLI2**	0,4	0,61	0
**β-CATENIN**	0	0,67	0
**LEF/TCF**	0	0,67	0
**RUNX2**	0	1	0
**SOX9**	1	0	0
**PTHRP**	0,77	0,09	0
**PPR**	0,77	0	0
**PKA**	1	0	0
**MEF2C**	0,04	0,49	0
**FGF**	0	1	0
**FGFR3**	0	0	0
**STAT1**	0,09	0,7	0
**SMAD complex**	0,19	0	0
**NKX3.2**	1	0	0
**ERK1/2**	0	1	0
**TGFβ**	0,4	0,61	0
**SMAD7**	0,19	0,66	0
**SMAD3**	0,26	0,19	0
**FGFR1**	0	1	0
**ATF2**	0,03	0,03	0
**NFκB**	0	1	0
**HDAC4**	0	0	0
**CCND1**	0,41	0,12	0
**DLX5**	0	0,27	0
**BMP**	0,28	1	0
**P38**	0,41	0,89	0
**GSK3β**	1	1	1
**DC**	1	0,33	1
**PP2A**	1	0	0
**AKT**	0	0,7	0
**PI3K**	0	0,7	0
**ETS1**	0,18	0,19	0
**RAS**	0,1	1	0
**IGF-1R**	0,31	0	0
**MSX2**	0,45	0	0
**δ-EF1**	0,13	0,83	0
**ATF4**	0,7	0,7	0
**HIF-2α**	0	1	0

**Fig 2:**
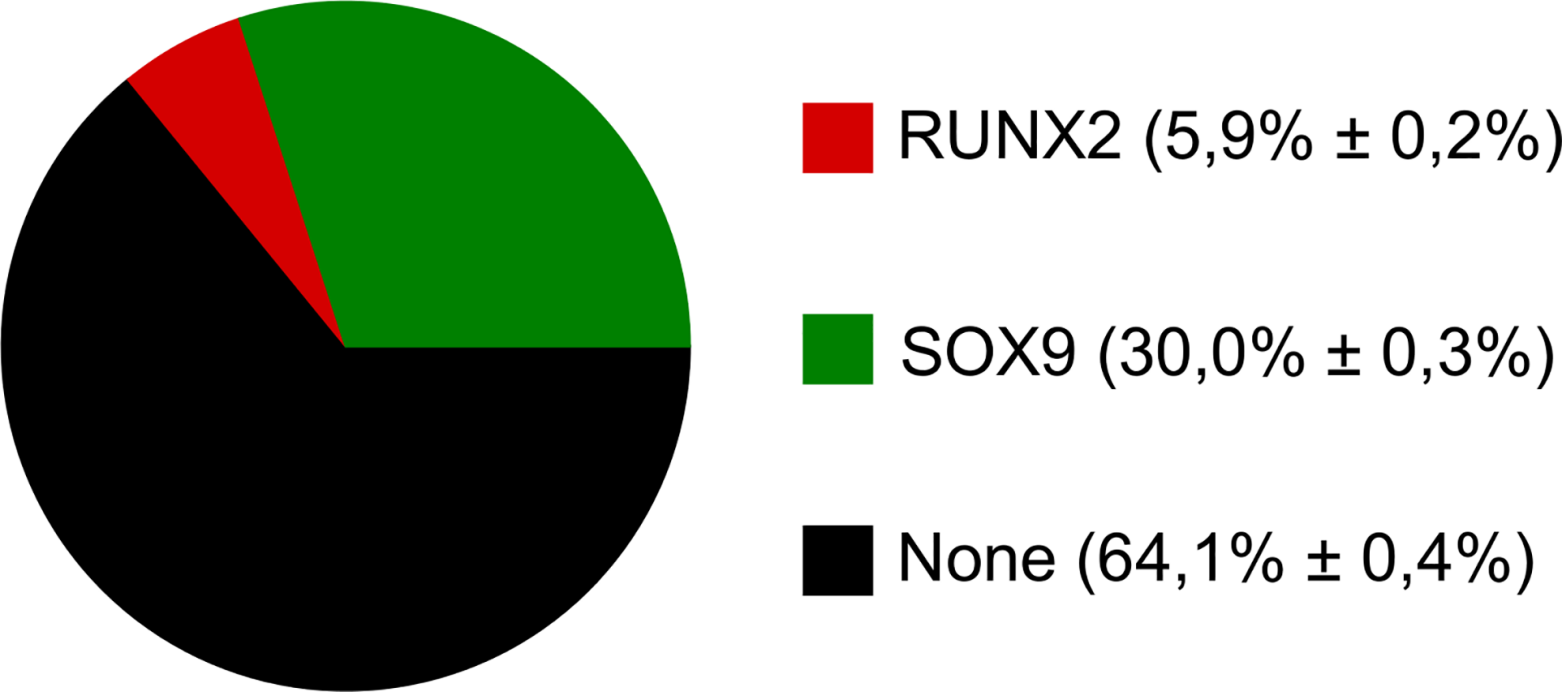
The estimated size of the attractor basins for the wild type network.

We then determined the probability that a random initial state will end up in each attractor as an estimation of the size of the attractor basin. We performed three runs of 10000 initial states and found that the ‘None’ basin of attraction was the largest one, absorbing about 64% of the initial states. The second largest attractor was found to be the SOX9 attractor with approximately one third of the states settling there. The RUNX2 attractor proved to be the smallest, at about 6% of states. Thus the robustness in canalisation of the RUNX2 stable state for the case of random initial conditions appears to be higher than that of SOX9. The results of this Monte Carlo analysis are given in Fig 2.

**Fig 3:**
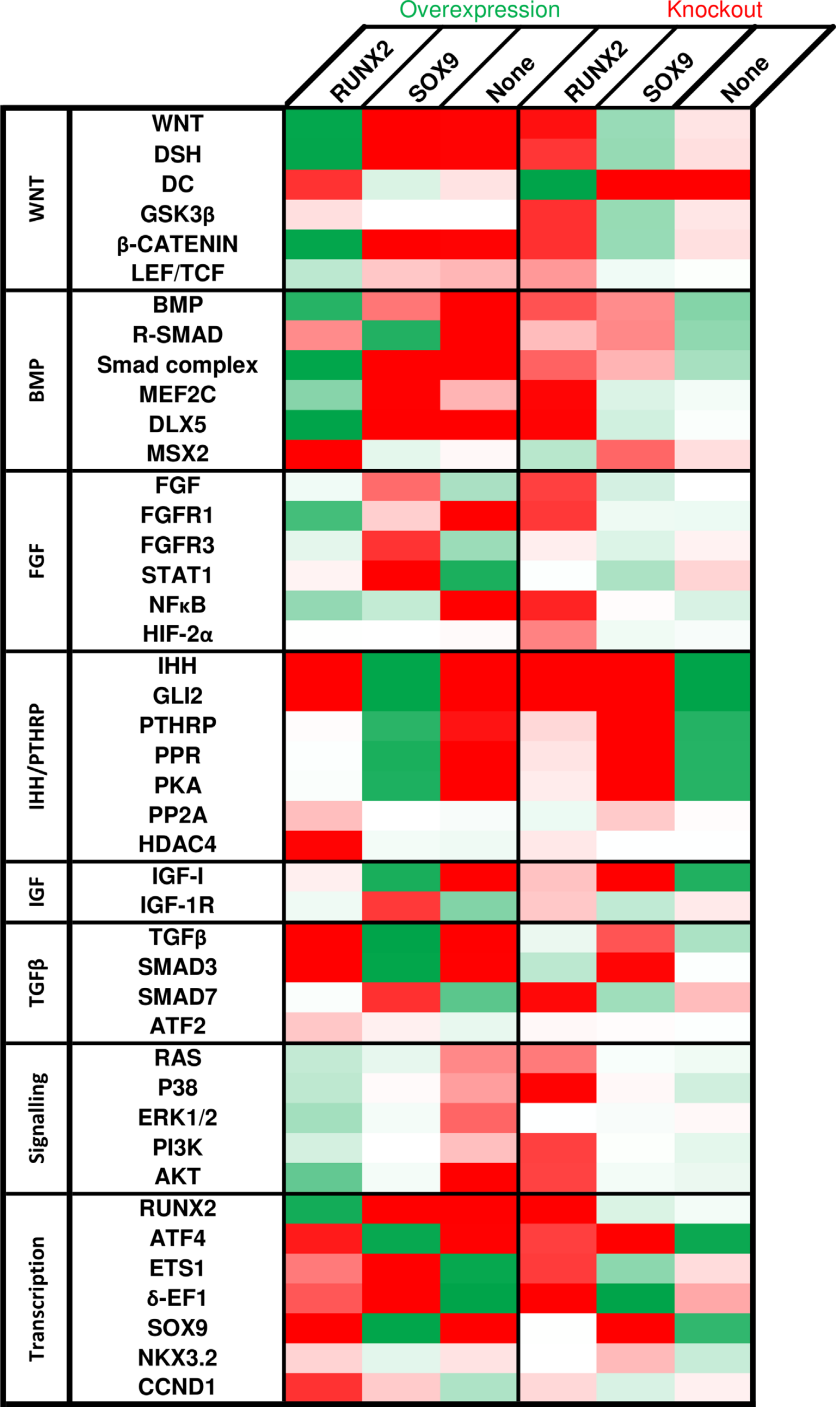
Predicted effect on the overexpression and knockout of the network’s genes using Monte Carlo analysis. Overview of changes in the size of attractor basins upon perturbation of the nodes. Both overexpression (first three columns) and knockout (last three columns) are shown. Each column is colour-coded, up regulation effects are green and down regulation effects are red. For instance, the first row for the first column indicates that WNT overexpression results in an increase (green) of the attractor basin of the RUNX2 attractor. Different shades of the colours indicate the intensity of the change. No change is indicated by white.

The results of the Monte Carlo analysis to estimate the influence of node perturbations on canalisation are shown in Fig 3. This analysis assesses the robustness of the stable states under perturbation by initializing at random states, with one node fixed, to estimate the size of their respective attractor basins. Fig 3 is divided in two main parts, the first one shows the effect of the node when it is overexpressed, whereas the second part shows the results of a knockout. The changes in the attractor basin size are shown for each of the network’s nodes. For an easier interpretation, the results are colour-coded with increasingly deep shades of green or red to indicate stronger increases or decreases, respectively. A basin with no change is depicted as being white.

Several factors were capable of maximizing the canalisation of the SOX9 attractor. Conversely, the knockout of many factors removed the RUNX2 basin entirely, indicating that the SOX9 attractor generally has a better canalisation than the RUNX2 attractor in this model. The primary factors that amplified the SOX9 attractor basin are IHH, GLI2, TGFβ, SMAD3, ATF4, IGF-I, PPR, PKA and PTHRP in order of descending magnitude. The factors that were required for RUNX2 canalisation, as in their absence no RUNX2 attractor basin is found, are IHH, GLI2, MEF2C, SMAD7, DLX5, P38 and δ-EF1. We did not detect any attractors where SOX9 and RUNX2 were both active, with the exception of the gain of function of R-SMAD and to a small extent that of BMP. Particularly, in the dominating RUNX2 (100%) attractor of the perturbed network, a small amount (20%) of SOX9 activity is present.

**Fig 4:**
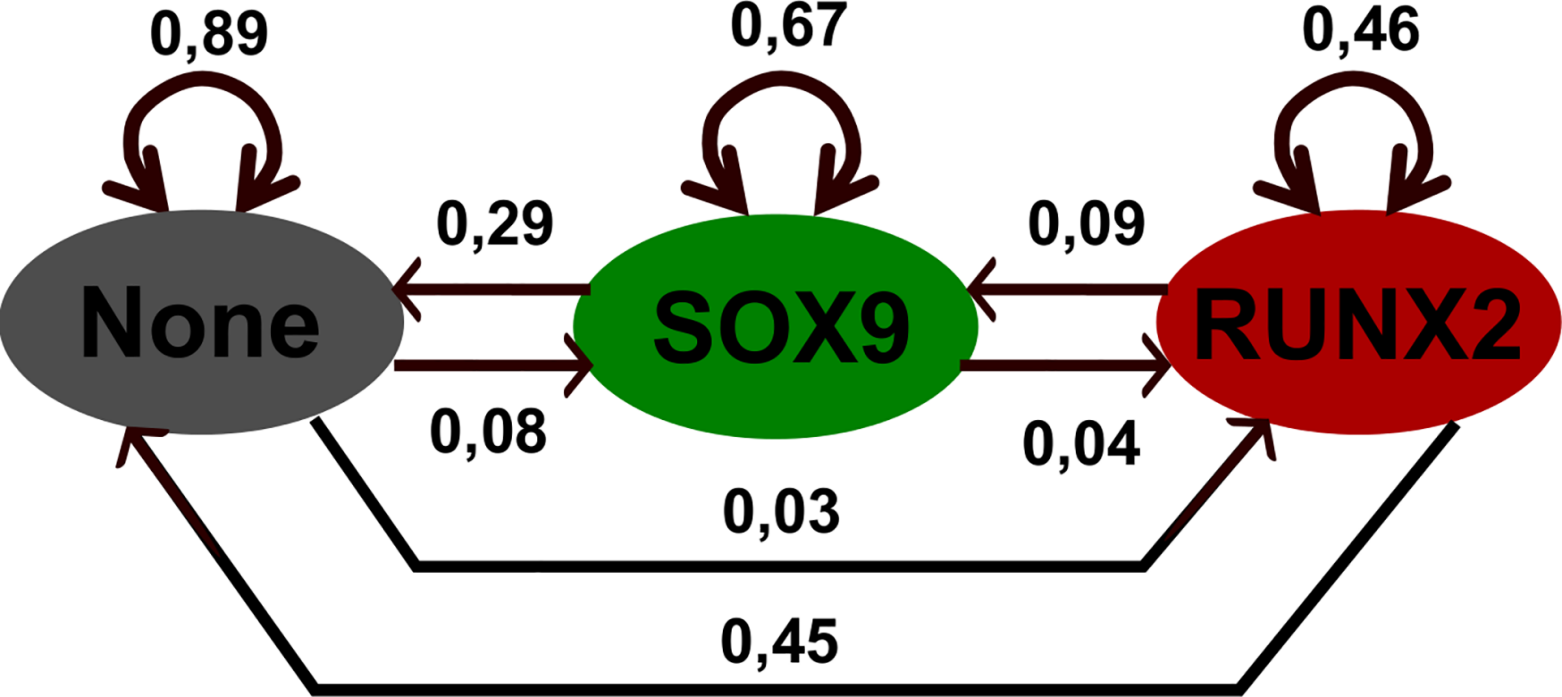
Markov Chain representation of the network. Circles represent steady states and the edges transitions between them. The corresponding transition probability is given for each edge.

**Fig 5:**
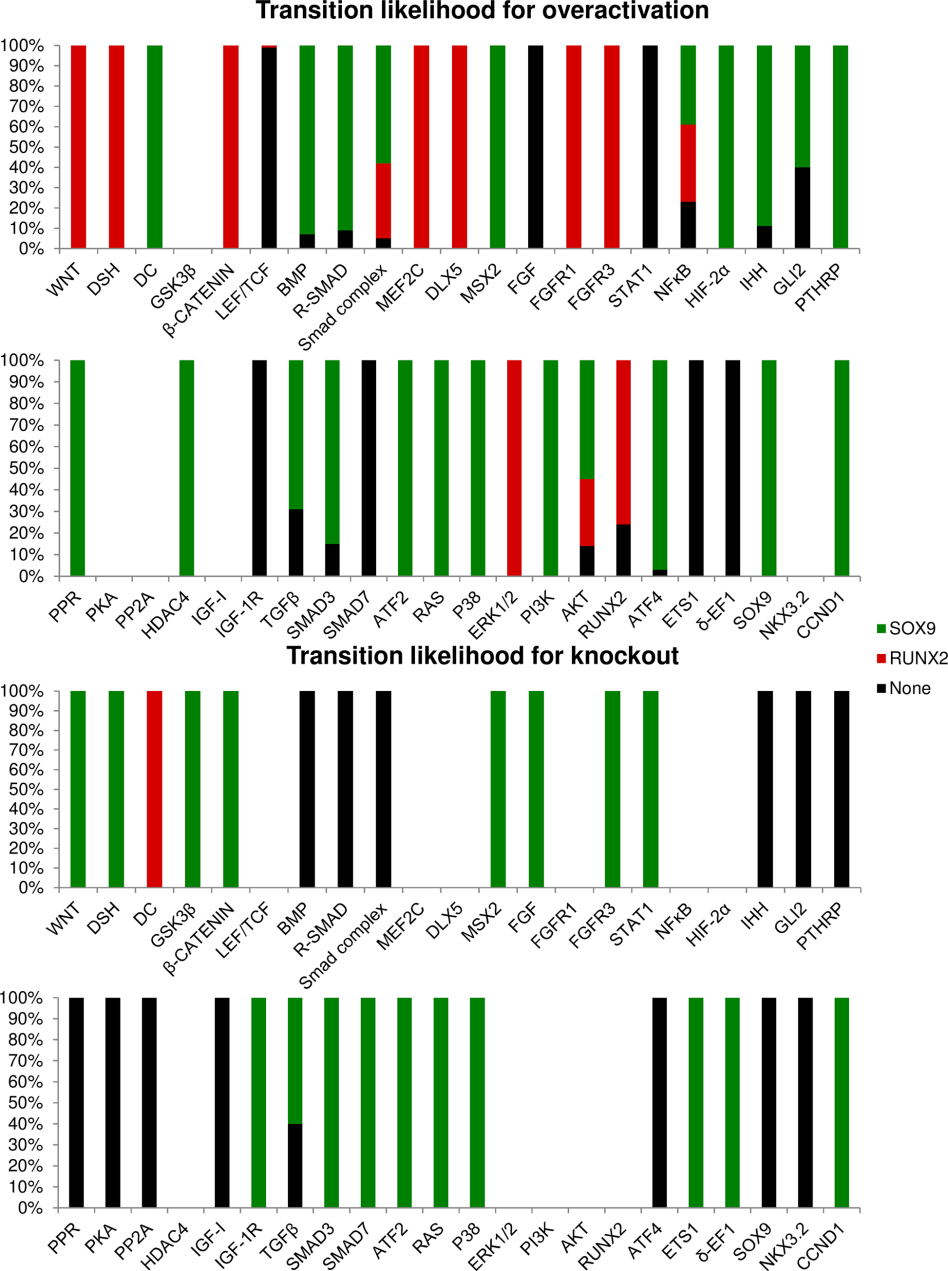
The effect of perturbations in the SOX9 attractor. For each node, the average outcomes for three times a hundred perturbations are shown in this Fig The colour code indicates the attractor the system settled in after perturbation. Nodes at maximal value are excluded for overactivation and nodes with zero activity are excluded for knockout.

Next, we investigated the stability of the attractors to perturbations in single nodes (in model 1). This is achieved by perturbing a node to maximal/minimal value in the steady state itself. The duration of the perturbation was taken so that a further increase had no effect on the results. The outcome for individual states is summarised in Fig 4. In this analysis the ‘None’ attractor emerges as the most stable with 89% of perturbations returning. The ‘None’ state has a higher resistance to perturbation than the SOX9 steady state. The most likely transition between states is that between RUNX2 and ‘None’ at 45%, followed by the transition of SOX9 to ‘None’ at 23%. The transition between SOX9 and RUNX2, i.e. the hypertrophic switch, occurs in 4% of perturbations. These transitions allow for the calculation of the distribution of attractors that remains constant in time, i.e. the stationary distribution. In this steady state ‘None’ holds about 75% of states, SOX9 19% and RUNX2 6%. Hence we expect the ‘None’ attractor to be adopted in the majority of cells under this (large) perturbation regime. In this analysis, the transition from SOX9 to ‘None’ or RUNX2 are intuitively most relevant for cartilage biology. The former might model dedifferentiation and the latter represents the natural developmental process or spurious hypertrophy. For these transitions, the effect of individual factors can be found in Fig 5.

Of the perturbations that induce hypertrophy, all but one are overactivations. Many of the knockouts lead to a loss of SOX9 phenotype without inducing RUNX2. For instance, loss of growth factors like IHH, IGF-I and PTHrP is predicted to lead to a loss of phenotype. Overactivations, such as those of R-SMAD and Smad complex, tend to have a more ambiguous effect. The perturbation can lead to a transition to all attractors, depending on the dynamics (i.e. which nodes are updated first). Several nodes from the BMP, WNT and FGF pathways can induce a hypertrophic switch at maximal perturbation. However, a majority of transitions end up in the more stable ‘None’ attractor. The magnitude (or duration) of the perturbation of course affects the outcome. For smaller perturbations (up to 4%) all attractors are stable.

The node that can perturb an attractor with the smallest increment is SMAD3, whose perturbation results in a transition from the RUNX2 to the ‘None’ attractor. SMAD3 directly inhibits RUNX2 activity and furthermore activates CCND1, another inhibitor of RUNX2. The next most effective nodes are IHH and GLI2, that similarly cause a transition from RUNX2 to ‘None’. These nodes play a vital role in the network as they act upstream of many of the network’s growth factors (in particular WNT, BMP, PTHRP and TGFβ). Hence, with their diminished activity, the ‘None’ attractor is reached, as can also be seen in the canalisation analysis (Fig 3). In fact, these nodes are also the most efficient in inducing a transition from the SOX9 to ‘None’ attractor. Nodes associated with the BMP pathway (BMP and R-SMAD) are the most efficient effector of the hypertrophic transition. As seen in S2 File, the results of this analysis, particularly the attractor that is reached after perturbation, are more sensitive to parameter values than those of the canalisation analysis.

The growth factors that most effectively increased RUNX2’s attractor basin were WNT and BMP. For these pathways, we assessed the response of SOX9 and RUNX2, the model’s output, to simulation with the relevant ligands as well as the combination of the two. In addition, mRNA expression of other relevant transcription factors were assessed, i.e. *DLX5* and *MEF2C*. We selected these factors because their expression level is directly relevant for the network’s dynamics. Downstream signals of WNT were not measured, as phosphorylated β-CATENIN not its mRNA, are indicative of WNT activity. As a control, the mRNA expression of *ID1* (for BMP) and *AXIN2* (for WNT) was examined.

**Fig 6:**
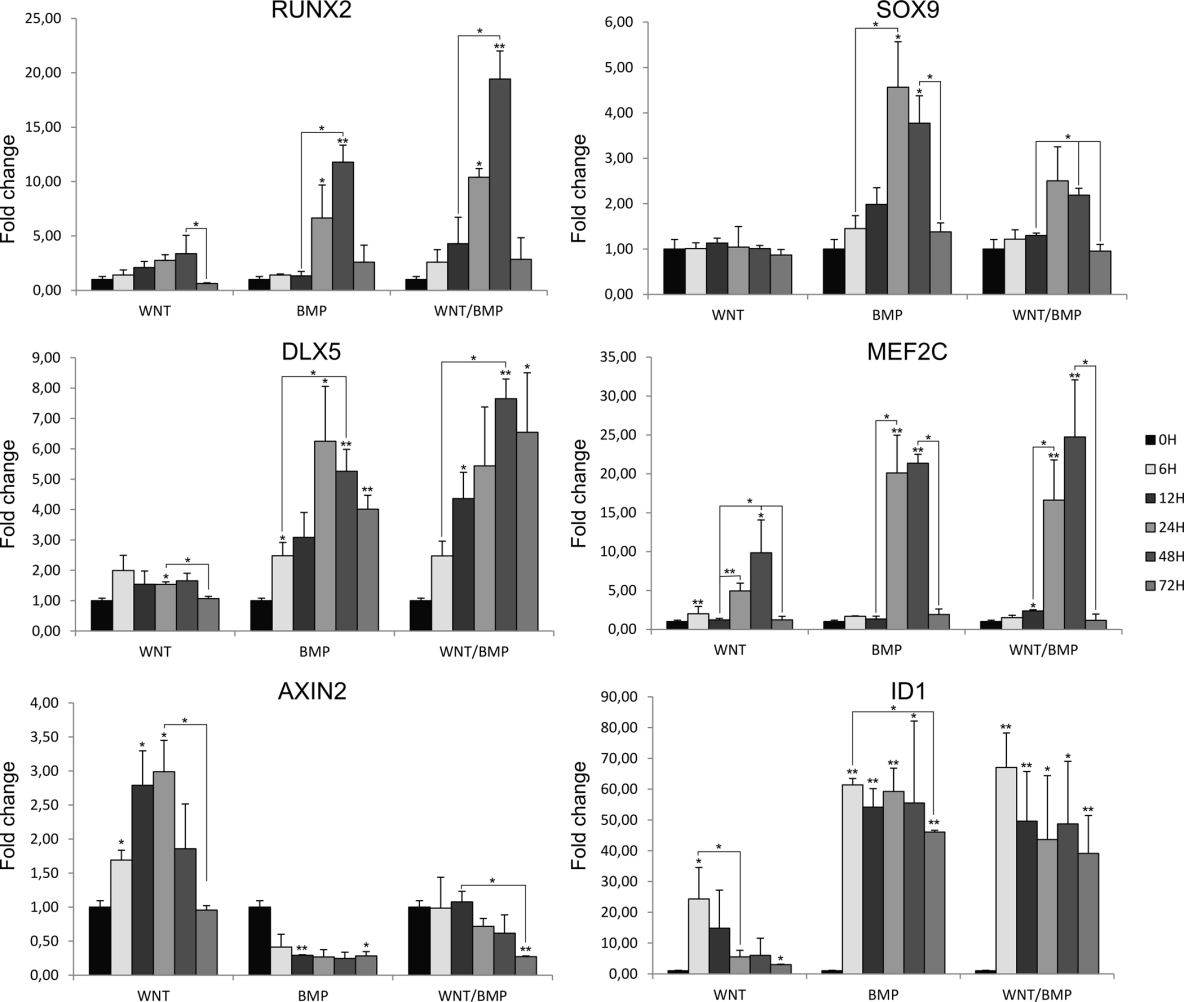
Gene expression for DLX5, MEF2C, RUNX2, SOX9 and control genes. A. Fold changes in expression of RUNX2, SOX9, DLX5, MEF2C, AXIN2 and ID1 are shown for different time points in the WNT, BMP and WNT/BMP conditions. Error bars indicate the standard deviation. The results were analysed by paired t-tests (corrected for multiple testing using the Benjamini-Hochberg procedure). *: p < 0,05. **: p < 0,01.

The results of WNT and/or BMP stimulation of human periosteal cells are shown in Fig 6. The cells were stimulated with BMP2, Wnt3a or a combination, and at specific time points mRNA was extracted for analysis by qPCR. These time-points were chosen to differentiate between direct and indirect effects of WNT and/or BMP stimulation. These experimental results can be compared to *in silico* experiments. The results of individual activations of WNT and BMP can be found in Fig 3. The double overactivation can be found in Table 2. In Table 2, we selected the 48h time point for the comparison since maximal expression was attained at this time in most of the conditions.

**Table 2:** Influence of overactivation of WNT and/or BMP on attractor canalisation. Model 1 is the network as shown in Fig 1. Model 2 is the network where the interaction BMP →MEF2C (slow) was replaced by the interaction WNT→MEF2C (slow). In model 3, the interaction BMP →MEF2C (slow) was removed, leaving only RUNX2 to regulate expression of MEF2C mRNA. For each model, the relative size of the attractors basins of RUNX2 and SOX9, and ‘None’ attractors is shown for the overactivation of WNT and/or BMP.

	Model 1			Model 2 (WNT→MEF2C)			Model 3 (RUNX2→MEF2C)		
	RUNX2	SOX9	None	RUNX2	SOX9	None	RUNX2	SOX9	None
**Wild type**	6%	30%	64%	6%	29%	65%	5%	30%	65%
**ΔWNT+**	+93%	-30%	-63%	+94%	-29%	-65%	+95%	-30%	-65%
**ΔBMP+**	+80%	-16%	-64%	+24%	+35%	-60%	+33%	+28%	-61%
**ΔWNT+/BMP+**	+94%	-30%	-64%	+74%	-9%	-65%	+57%	-7%	-50%

The results of this experiment support the activation of RUNX2 by BMP and WNT, but show that the response of SOX9 to these growth factors is not reflected well in the current model. In the standard model, both RUNX2 and the SMAD complex regulate MEF2C transcription. However, addition of BMP to the cells shows that MEF2C is upregulated 20 hours after BMP treatment, at the same time as its upstream regulator, RUNX2. From this we assume that *MEF2C* expression is unlikely to be directly regulated by BMP, but rather is regulated indirectly by another factor than SMAD complex and/or RUNX2. In addition, we observe an increase in *MEF2C* expression already at 6 hours after addition of WNT3a, indicating that WNT signalling plays a role in the regulation of *MEF2C* expression. Interestingly, combined treatment with WNT and BMP did not result in higher *MEF2C* expression. We therefore opted to explore the dynamical behaviour of a set of plausible networks by varying the topology upstream of *MEF2C*. In one alternative model (Table 2, Model 2), we selected a topology where *MEF2C* mRNA is downstream of WNT (LEF/TCF) signalling, rather than SMAD complex [66]. As the upregulation of *MEF2C* in response to WNT signalling is small (1.5 fold after 6H) and limited *MEF2C* upregulation is seen before *RUNX2* is upregulated, a second alternative topology is selected (Table 2, Model 3) where only RUNX2 is retained upstream of *MEF2C* mRNA. For comparison, results for *in silico* simulations of WNT and/or BMP overactivations can be found in Table 2. Compared to the original network, both Model 2 and Model 3 capture the effect of overactivations on SOX9 stability more accurately. Since the response of *MEF2C* to WNT stimulation is weak, Model 3 would be the preferable topology for this particular cell type.

An increased understanding of the regulatory mechanisms operating in hypertrophy can pinpoint therapeutic targets in cartilage-related diseases and instruct approaches in bone and cartilage tissue engineering. For example, the results pertaining to the stability of the RUNX2 attractor basin can be used to suggest therapeutic targets for preventing hypertrophy. We have surveyed available literature on these suggested therapeutic targets. Table 3 assesses the potential of the nodes whose perturbation decreases the size of the RUNX2 attractor basin, and does not decrease the SOX9 attractor basin size, as therapeutic targets in osteoarthritis. To assess this potential, we searched literature to check if the effect of a knockout could confer protection to OA. The expected phenotype was observed for 5 factors, a reverse effect was found in 2 cases and no knockout was found for 8 factors. In the case of FGF we found conflicting effects on hypertrophy depending on the type. For one the reverse cases (GSK3β), a double *in silico* KO would be more appropriate since GSK3β is also a part of the destruction complex (DC). This double KO did match literature results.

**Table 3:** Effect of knockout on OA disease model. *The first and second column contain the effect of a knockout in the attractor basin*. *We check whether a knockout (either through the use of a small molecule inhibitor or by a genetic KO) indeed confers protection to OA*, *indicated as ‘protective’*. *Sometimes a knockout is seen to aggravate symptoms*, *indicated as ‘reverse’*. *Finally*, *cases where no knockout was found*, *are marked by ‘absent’*. *Sometimes indirect support*, *namely that the protein’s constitutive activation entails hypertrophy*, *is found*. *Papers indicating this are referenced after the ‘absent’ tag*. *For SMAD7*, *one study found increased Smad7 expression was accompanied by a reduction in osteophyte formation* [67]. *In the network model, *an insulation of GSK in the destruction complex from cytosolic GSK is assumed*. *In the case of a knockout this would not hold*. *Hence a double mutant*, *with a knockout of both destruction complex (DC) and GSK*, *constitutes a more appropriate simulation*.

Knockout of	Effect on RUNX2	Effect on SOX9	OA effect?
**WNT**	-	+	protective[68]
**DSH**	-	+	absent
GSK3β (double KO*)	+	-	reverse*[69]
**β-CATENIN**	-	+	protective[70–75]
**LEF/TCF**	-	/	absent[76,77]
**DLX5**	-	/ to +	absent[78,79]
**FGF**	-	/ to +	reverse [80,81], protective[82,83]
**FGFR1**	-	/ to +	protective[84]
**NFκB**	-	/	reverse[85,86]
**SMAD7**	-	+	absent[67,87,88]
**RAS**	-	/	absent[89]
**P38**	-	/	protective[90]
**PI3K**	-	/	absent[91]
**AKT**	-	/	protective[92]
**ETS1**	-	+	absent
**δ-EF1**	-	+	absent

## Discussion

Great strides in our understanding of cartilage biology have been made in the past 2 decades, supported by tried techniques like immunohistochemistry, ever more specific mouse models such as cre-lox systems and high-throughput in vitro assays alike [93,94]. As this data accumulates in the wake of the field’s progress, it becomes increasingly harder to glean insight by human intuition alone, making computational approaches that can smartly reuse this data all the more indispensable [95]. Therefore we applied a qualitative approach that employs this readily available data, allowing for a first cursory exploration of the regulatory network in chondrocyte differentiation as a whole. This regulatory network and accompanying insights in cartilage differentiation can be used to inform cartilage and bone tissue engineering approaches and suggest treatments in differentiation-related cartilage pathologies. For the specific case of osteoarthritis, several suggested therapeutic targets could be corroborated and novel targets could be suggested.

In this work, we have employed two dynamical measures to gauge phenotypic robustness, i.e. the robustness of canalisation and the robustness to perturbation. Other structural measures, such as the simple path measure [96], or topological features, such as betweenness [61], could also be employed [97]. However, we focused on robustness of canalisation and to perturbation as they take into account the dynamical behaviour of the network, such as the effect of changes on stable states.

In our analysis we equate stable states in the model to cell types. In fact, most cellular networks are in a state of equilibrium, especially in adult tissues. We therefore assumed that stable cell types are defined by a stable mix of transcription factors that correspond with a stable state (or an ergodic set of attractors [98]) of the network. The view that an attractor of the network equates to a cell type already surfaces in Waddington’s vision of an epigenetic landscape and was brought to fruition in the early genetic nets introduced by Kauffman [63,99,100]. Indeed, distinct genome-wide expression patterns corresponding to known cell types provide empirical support to the concept of attractors, though the attractor itself may be defined by a much smaller set of transcription factors and their respective interactions [101–103]. This idea of a stable cellular phenotype being determined by a fixed set of transcription factors is exemplified by the induction of pluripotency from a range of highly differing starting populations through the effect of only four transcription factors (Yamanaka factors) in the formation of iPS cells [104]. The idea is vindicated to a greater extent as more examples of transdifferentiation due to reprogramming are brought to light, indicating that other cell types also rely on a limited core of transcription factors.

Reprogramming occurs in the presence of strong and prolonged stimuli. Indeed, in order to have an effect on cell behaviour a stimulus should be sustained in time and significant in amplitude. To ascertain that this premise holds true for the chondrocyte gene network we applied smaller stimuli to the network in the form of small perturbations. Our results show that for a small perturbation all states are robust and no transition between states is observed. In essence, the state is kept in the attractor basin by the positive feedback loops that reinforce and conserve the cellular phenotype and thus serve as an irreversible switch that is insensitive to noise. After the stimulus is removed the cell quickly falls back to the attractor. This indicates that the attractors of the network model can indeed remain established in the face of noise, which is unavoidable in an *in vivo* setting.

### Canalisation analysis

The dominant state in the canalisation analysis is the ‘None’ state. Three factors however limit the relevance of this result. Firstly, we have analysed the chondrocyte network in isolation, in the absence of a source of external growth factors. As seen readily in Fig 3 the fixing of growth factors at a certain value mostly abolishes this basin, with the exception of FGF. The regulatory network, modelled at the single cell level, is limited to autocrine growth factors. In contrast, cells from the growth plate rely on paracrine growth factors that are made available by their neighbours (e.g. the periost) or stored in the matrix. From this perspective, the behaviour of a population of cells, each driven by the regulatory network, would be interesting to investigate and would come closer to biological reality by adding an additional layer of cellular communication through diffusion of signals. Secondly, neither in development nor in adult tissue one would expect to start from a blank slate, meaning that starting positions closer to the ‘None’ attractor have less biological relevance. Lastly, the dominance of the ‘None’ state is also affected by the saturation factor. This factor determines how easy it is for a node to be activated if it has multiple inputs. A higher saturation factor increases the activity of many nodes and consequently decreases the size of the ‘None’ basin. In this sense, the dominance of the ‘None’ basin is not robust. In our analysis of the unperturbed network we did not detect any states where both SOX9 and RUNX2 are active, though transcripts and proteins of these transcription factors have been detected together in a single cell [105]. However, it is unsure whether the transcription factors are truly active and whether the state is truly stable or merely transient. For some perturbations (e.g. R-SMAD activation), it is indeed possible for both transcription factors to be active.

The results of the Monte Carlo analysis (Fig 3) are sometimes asymmetric in the sense that the effects of a knockout do not necessarily mirror the effects of overactivation. One example is the node IGF-I, whose activation is detrimental to the size of the RUNX2 attractor basin, but its absence does not negatively impact the size of the basin. Another example is PP2A, whose knockout is effective in destabilizing the SOX9 state, whereas its overactivation does not have a significant effect on SOX9 state’s stability. Conversely, the overactivation of HDAC4 increases SOX9’s basin, but its knockout has no effect. This seems to indicate that at least in some cases factors (such as ERK1/2 and HDAC4) that are important or required for the induction of a phenotype, i.e. whose overactivation increases the basin of attraction of a certain state, might not be important in maintaining it, i.e. their knockout does not decrease the basin of attraction to a same relative extent. Conversely, factors (such as PP2A) that are shown to be crucial for maintenance of a stable state may not be efficient in the induction of it.

Discrepancies with the obtained results can be found in literature. For instance, Qiao et al. [106] show that IGF-I has a positive effect on RUNX2 DNA binding, whereas the effect on canalisation in our results is very limited. Several potential explanations may be offered to interpret the difference. Firstly, the network combines data from a large amount of studies. Hence, while the interactions of described in Qiao et al. [106] were included in the model, a larger amount of positive interactions with SOX9 dominate the effect of IGF-I. Secondly, the canalisation analysis incorporates a wide (random) variety of situations, and the positive effect of IGF-I activity may depend on particular circumstances (e.g. proximity to the RUNX2 attractor) that represent a relatively small part of the state space. Lastly, the model only takes into account topology, and varying the strengths of interactions may change the results of the analysis. This last effect is limited however, as can be seen in S2 File.

### Perturbation analysis

The SOX9 attractor seems to be more proximate to the ‘None’ attractor and consequently has the highest chance to transition there in the face of perturbation. In keeping with Waddington’s metaphor of an epigenetic landscape the smaller SOX9 basin is only separated from the larger one corresponding to the ‘None’ attractor by a small crest whereas more impulse from external signals is needed to traverse the larger distance to the small RUNX2 attractor. One should also note the directionality between the SOX9 and RUNX2 attractor, it is much harder to go from SOX9 to RUNX2 than vice versa, which does not correspond to the sequence of events in development. Several observations correspond with a limited stability of the SOX9 attractor. First, the chondrocytic phenotype is notoriously difficult to keep intact *in vitro* as the cells rapidly dedifferentiate and lose expression of vital cartilage components [107–109]. Secondly, for many cell types (such as ATDC5s, mesenchymal stem cells and growth plate chondrocytes) and tissue engineering applications, it has proven challenging to maintain a purely chondrocytic phenotype, instead the developmental chain is continued by the process of hypertrophy. Thirdly, it was shown that the maintenance of the cartilage phenotype is dependent on secreted antagonists [110,111]. For this reasons the lower stability of the RUNX2 attractor compared to the SOX9 attractor in both the perturbation and canalisation analysis may be unrealistic. Hence, the network might be improved by imposing constraints on the attractor basin sizes (or transitions) to ensure a correct developmental path, as was done by Zhou et al [112].

Note that the perturbation analysis is affected by changes in the saturation factor (see S2 File). Particularly, the dominance of the SOX9 attractor ensures that less transitions take place. Many of the nodes that achieved a transition to the ‘None’ attractor are no longer effective, with the exception of the PTHRP pathway. This includes IHH and GLI2, as the higher value of the saturation ensures the sufficient growth factor activity is present. The transition from SOX9 to RUNX2 is less affected. Indeed, this transition is still most efficiently induced by activation of the BMP pathway. As the conclusions from the canalisation analysis proved to be more resilient to changes in the saturation factor, these have been used to interpret the *in vitro* experiment and suggest therapeutic targets in ectopic hypertrophy.

### Adaptation of network topology

To illustrate the application of mechanistic models and their use in substantiating hypotheses, the model’s output was compared to experimental data for the specific cases of WNT/BMP stimulation. We used a heuristic approach to adapt the topology of our network to better capture the result of a series of experiments. Besides the network’s output of *SOX9* and *RUNX2* some relevant downstream effectors such as *MEF2C* and *DLX5* were measured. These could be used as an indication of where network topology may be wanting. Specifically, we focused on the upstream control of *MEF2C*, as the experimentally observed response of *DLX5* did not contradict network topology. In literature, *MEF2C* is described as an indirect target for BMP signalling in cardiomyocyte precursors through GATA transcription factors [113]. In the absence of NKX3.2, BMP signals can likewise induce these factors in the developing limb [114]. However, the activation of *Mef2c* by BMP signalling was not yet demonstrated for the osteochondral lineage. Indeed, our data shows that *Mef2c* was not transcribed downstream of BMP signalling, prompting us to propose two alternative topologies more commensurate with MEF2C data. As *Mef2c* is regarded as a target of WNT signalling [66] and given the (weak) early *Mef2c* expression in the WNT condition, an alternative topology with WNT upstream of *Mef2c* was evaluated. A second more parsimonious topology retained RUNX2 upstream of *Mef2c* [115].

As seen in Table 2, these topologies resulted in a better match to SOX9/RUNX2 dynamics. In contrast with the original model, where combinations of WNT/BMP only increase RUNX2 stability, the adjusted models showed a distinction in *SOX9* dynamics. In accordance with the trend of the *in vitro* experiment, the *in silico* results showed no *SOX9* activation in the case of WNT signalling, a high activation upon BMP stimulation and the WNT/BMP combination resulted in an intermediate expression level. Note that due to the high number of included interactions, the change in one particular interaction did not greatly affect the overall behaviour of the included nodes in canalisation, as seen in S2 File.

Through comparison with the model, changes in expression levels of fate determining transcription factors such as SOX9 and RUNX2 are framed in the larger context of the regulatory network. Hence, the network's topology and predictions of dynamics ultimately constitute a useful tool to develop mechanistically supported hypotheses and help in providing dynamically plausible and coherent explanations for *in vitro* observations. The context (and summary of literature knowledge included) provided by the model allows for the formulation of rigorous hypotheses on the effect of particular perturbations *a priori*, and can vet alternatives to resolve discrepancies *a posteriori*. In this light, the model represents a synthesis of information abstracted from developmental biology studies that can be adapted, through comparison with experimental results, to a related and more clinically relevant application in hPDCs.

Next to adapting the topology itself, an alternative way for adjusting the model is to change interaction weights to better match the experiment’s outcome. Ideally, discrepancies with experiments (or growth plate expression profiles) should be resolved by an automated procedure, avoiding the somewhat arbitrary selection of changes in topology (or interaction weights) associated with manual curation. In this way, a more systematic approach to refine predictions with experimental data could be incorporated in our model framework.

Since the experiment was limited to a single dosage of growth factors, it was not possible to match the relative strength of the growth factors to the normalised variables of the model. For instance, the induction of RUNX2 (and other measured factors) was much stronger for BMP than for WNT. However, a higher dose of WNT growth factors may alleviate or reverse this trend. Hence, the analysis was limited to a qualitative assessment of the growth factor’s influence, and no attempt was made to capture their relative strengths, for example by varying interaction weights. As such, for the overactivations of Table 2, we have fixed the node’s activity at 100%. As this results in the disappearance of the ‘None’ basin, the SOX9 basin was slightly smaller than the wild type baseline for the BMP and WNT/BMP overactivations (Table 2, Model 2 and 3). For lower levels of activity, the SOX9 attractor basin does exceed that of the wild type situation (and is larger for BMP than for BMP/WNT), more in line with the *in vitro* observations.

### Limitations

The current study uses SOX9 and RUNX2 as biomarkers for proliferation and hypertrophic differentiation, respectively. While the use of these genes as biomarkers is in accordance to conventional wet-ware approaches, we have only used them as examples. In fact, any gene node present in the network can be analysed in a similar fashion, allowing fast and straightforward predictions of envisioned stimuli on e.g. anabolism, catabolism, (indirect) crosstalk between signalling pathways and production of trophic mediators. The equation of SOX9 with a proliferative chondrocyte and RUNX2 with a hypertrophic one is easily falsified by a few examples to the contrary. For example, limb bud cells are all SOX9 positive, while only a fraction of them will ultimately form stable cartilage. On the other hand, mouse studies show that RUNX2 is not the sole proprietor and guarantee for robust hypertrophy [116,117]. Moreover, osteoblasts also rely on RUNX2 as a master gene and given the high similarity in expression profile with hypertrophic chondrocytes, it is likely that their ‘gene programs’ are one and the same [59]. In fact, an argument could be made that the first measure, i.e. canalisation starting from random initial conditions, is more reflective of the earlier developmental choice between an osteoblastic and a chondrocytic differentiation route [118]. Hence we used hPDCs in our *in vitro* experiments, since they are a multipotent cell type where SOX9 and RUNX2, as in growth plate chondrocytes, take up a role in fate determination [119,120]. Furthermore, their differentiation is governed largely by the same pathways seen in development [121,122]. Arguably, limb bud cells would constitute a more suited cell type to corroborate the model’s results, as most of the interactions were derived from studies on the fetal growth plate. However, this cell type is not readily available and, as they are already positive for SOX9, may not reflect the canalisation analysis.

The main difference between the osteoblastic and chondrocytic phenotype may not actually be the core transcription network as modelled here but rather the developmental path that was followed to get there. In the case of an osteoblast, a direct route to RUNX2 expression is taken, whereas in the hypertrophic chondrocyte, SOX9 and a set of related transcription factors are activated first. Due to slow degradation, lingering proteins then are responsible for the difference between the two [59]. As such, the resistance to perturbation of the SOX9 attractor is probably the superior measure to assess the stability of the chondrocyte. This is not a moot point, as results based on robustness in canalisation can significantly differ from those of the perturbation analysis (detailed in S2 File).

Though our model is of relatively large size, many pathways are yet omitted from the network model. We have focused mainly on paracrine signalling and information from developmental biology, leaving out for example hormonal regulation and mechanoregulation which may have profound influence on the phenotypic stability of the chondrocyte [123–125]. Similarly, we may also underestimate the stability of the cartilage since we have not included metabolic effects such as hypoxic conditions, cartilage being an entirely avascular tissue. Indeed, hypoxia was shown to effectively inhibit hypertrophy indicating its importance [126]. Additionally, our model has focused on regulatory events in the growth plate, and hence the application to the biology of permanent cartilage or fracture repair is indirect. A previous integrative modelling approach focused on short term signalling events and included many inflammatory pathways [127]. They also suggested mechanisms in their model which could induce hypertrophy, of which MEK1/2 (upstream of ERK1/2) and FGF2 overlap with our model. These factors were indeed shown to induce hypertrophy. Nevertheless, the presented model still includes a significant amount of determinative factors in chondrogenesis, and, we believe, captures a more comprehensive view of hypertrophic differentiation than any other model to date.

### Relevance to tissue engineering and osteoarthritis

The results of our *in silico* screening can be regarded as a guidance for bone tissue engineering strategies. Indeed, any factor that enlarges the RUNX2 basin can be considered as an inducer of hypertrophy. Likewise, all factors that increase the SOX9 basin can be considered inducers of the cartilage fate. Factors that are required to maintain a SOX9 basin are a necessary condition for the formation of cartilage and their presence could be monitored as indicative of ‘healthy’ cartilage. In the case of ectopic hypertrophy, factors that maintain the RUNX2 basin represent putative therapeutic drug targets. Even if knockout of these maintenance factors may not succeed in reversing hypertrophy, reducing the activity of RUNX2 alone may be sufficient to provide a therapeutic benefit. For example, in OA RUNX2 is upstream of MMP13 whose downregulation can help in normalizing cartilage turnover [128]. Preferably, this same factor would increase the SOX9 attractor basin or at least not diminish it.

The model and the results of the *in silico* screening also provide many novel opportunities for the understanding and the development of new treatments for OA. Indeed, as a complicated multifactorial disease it is difficult to understand or predict how all the separately reported studies on OA interlace. To date, no disease modifying osteoarthritis drugs (DMOADs), including MMP inhibitors, showed significant impact on OA pathophysiology, in part due to this complexity. As such, our genetic network is ideally suited to untangle this complexity as it is sufficiently advanced to make such connections and interactions. For instance, it was recently suggested that HIF-2α is an important etiological factor in OA as it is a transcriptional activator of many genes crucial in endochondral ossification and thus should prove efficient in inducing ectopic hypertrophy [33]. However, our results show that HIF-2α is a very modest contributor to the onset of hypertrophy. This result is corroborated by the observation that hypertrophy is only modestly and transiently delayed in mice with a limb bud-specific knockout of *HIF-2α* [129]. Of course, the lack of importance in the induction of hypertrophy could be offset by the direct link between HIF-2α and catabolism through MMP13 and other catabolic factors [34]. Our model hence does not directly contradict the proposed etiological importance of HIF-2α, but suggests its effect is modest. Nevertheless, direct importance of HIF-2α in the downstream events of endochondral ossification ensures that any mutations increasing the activity of this gene, while not causative of the hypertrophic phenotype, can have a detrimental effect on cartilage homeostasis, thus exacerbating OA pathophysiology.

Additionally, the factors that diminish the hypertrophic basin of attraction were checked for their effectiveness in preventing OA in relevant literature. In other pathophysiological processes such as heterotopic ossification this list is equally valid, but the large body of literature documenting OA makes it the most practical choice. The wide range of biological processes reflected in a node’s activity, make a direct validation challenging. For example, many key nodes in our network reflect the active form of transcription factors, which is hard to measure in practice [20]. Furthermore, a permanent perturbation, as used in the canalisation analysis, is only possible by genetic manipulation. Any pharmaceutical agent, such as a small molecule inhibitor, would only engender a temporary perturbation [96]. However, the dynamics of short perturbations mostly follow the same trends as those of longer perturbations. Of 16 factors suggested to confer increased resistance against hypertrophy and consequently OA, 5 could be corroborated, 2 falsified, 1 was ambiguous (FGF18 does not have the predicted effect whereas FGF2 does) and 8 were not tested to our knowledge.

In conclusion, our network is a summary of information gleaned from a host of studies on chondrocyte differentiation. Moreover, it provides a qualitative framework in which advances in the field from ongoing and future experiments can be incorporated to further enhance its predictive power. This compendium allows for an *in silico* screening that can provide input for tissue engineering strategies and suggest candidates for intervention in disease. As such, this network complements experimental approaches and helps pave the way to a system-level understanding of chondrocyte differentiation.

## Supporting Information

S1 File References for interactions included in the model. The first and second column give the originating node and the target node respectively. The fourth column indicates the model system that was used in the referenced studies.
(PDF)

S2 File Discussion of the influence of the saturation factor. (PDF)

S3 File Full list of equations of the chondrocyte gene regulatory network. (PDF)

S4 File MATLAB files used to perform dynamic analysis. The files Mutant_Monte_Carlo.m and Perturbation_analysis.m were used to perform the Monte Carlo and the Perturbation analysis, respectively.
(ZIP)

S1 Table Primer sequences for RT-PCR. (XLSX)
