# Supplementary material for: A Qualitative Model of the Differentiation Network in Chondrocyte Maturation: A Holistic View of Chondrocyte Hypertrophy
Source: PLoS One. 2016 Aug 31;11(8):e0162052. doi: 10.1371/journal.pone.0162052 (PMC5007039; doi:10.1371/journal.pone.0162052)
Supplement: S1 File — The first and second column give the originating node and the target node respectively. The fourth column indicates the model system that was used in the referenced studies. (PDF) [file pone.0162052.s001.pdf]

Active node	Activated node	Reference	System
HDAC4/Smad3	Runx2	[1,2]	Mouse *in vivo* chondrocyte, primary calvarial mouse osteoblasts
HDAC4	Runx2	[3,4]	E17 cephalic chick sternum chondrocytes
Lef/Tcf	Runx2	[5]	NIH3T3 (mouse fibroblastic), C3H10T1/2 (mouse mesenchymal), MC3T3-E1 (mouse preosteoblasts)
Runx2	PI3K	[6,7]	ATDC5 (mouse chondrogenic cell line), C3H10T1/2 (mouse mesenchymal), MC3T3-E1 (mouse preosteoblasts)
Runx2	Akt	[6,7]	ATDC5 (mouse chondrogenic cell line), C3H10T1/2 (mouse mesenchymal), MC3T3-E1 (mouse preosteoblasts)
Akt	Runx2	[6,7]	ATDC5 (mouse chondrogenic cell line), C3H10T1/2 (mouse mesenchymal), MC3T3-E1 (mouse preosteoblasts)
Msx2	Runx2	[8]	C2C12 (mouse(pre)osteoblasts)
Msx2	Wnt2b	[9]	Promotor study
Gli	Wnt2b	[9]	Promotor study
Dlx5	Msx2	[8]	C2C12 (mouse premyoblasts)
Smad3	Sox9	[10,11]	Human MSCs, human chondrosarcoma cell line (SW1353)
Sox9	β-catenin	[12–14]	Mouse limb bud cells, murine primary chondrocytes , L cells (fibroblasts), CHO (chinese hamster ovary), HEK293 (Human embryonic kidney), COS1
β-catenin	Sox9	[13,14]	
IGF	PKA	[15]	mesenchymal chondrogenic cell line RCJ3.1C5.18
p38	MEF2C	[16]	Yeast 2-hybrid screen (Y2H) (human)
Smadcomplex	MEF2C	[16]	Y2H
Smad3	MEF2C	[17]	C3H10T1/2
IGF-IR	STAT1	[18]	Multiple including rat cardiomyocytes
Ras	ERK	canonical	

PP2A	ERK	canonical	
Smad7	Smad3	canonical	
ERK	Smad3	[19,20]	Mv1Lu/R1B-L17 (mink lung epithelial cells), EpH4 and EpRas (mouse mammary epithelial)
CCND1 (cdk2 ,-4)	Smad3	[19,21]	Mv1Lu
Smad3	ATF2	[22]	*In vitro* binding assay, Mammalian 2-hybrid assay
p38	ATF2	[22]	*In vitro* binding assay, Mammalian 2-hybrid assay
Akt	NFκβ	[23]	N1511 (mouse chondrocytic cell line)
GSK3β	HDAC4	[24]	MCF-10A human mammary epithelial cells) and NIH-3T3 (murine fibroblast cell line)
GSK3β	CCND1	[25,26]	NIH-3T3 cells
PP2A	HDAC4	[27]	*In vitro* binding assays
Msx2	Dlx5	[28–30]	*In vitro* binding assays, Y2H
p38	Dlx5	[31]	*In vitro* phosphorylation assay, C2C12 (mouse premyoblasts) and HEK-293 (Human embryonic kidney cells)
TGFβ	p38	[32]	Mouse primary sternal chondrocytes, chick articular chondrocytes
ERK	GSK3β	[33]	*In vitro* phosphorylation assay
GSK3β	DC	[34]	
PKA	GSK3β	[35]	*In vitro* phosphorylation assay (human)
ERK	DC	[33]	Diverse cancer cell lines (human)
PKA	PP2A	[36]	Mouse and chick limb bud cells
PI3K	Akt	canonical	
PP2A	Akt	[37,38]	3T3-L1 mouse adipocytes and others
Ras	PI3K	[39,40]	Chick sternal chondrocytes, *In vitro* assay
IGFI-R	PI3K	[41,42]	Primary human chondrocytes, rat growth plate
GSK3β	Ets1	[43]	In vitro phosphorylation assay (human)
ERK	Ets1	[44,45]	Mesencephalic neural crest cells (mouse), drosophila
BMP	Ras	[46]	C3H10T1/2 (mouse mesenchymal), C2C12

			(mouse premyoblasts)
IGF-I	IGF-IR	canonical	
Dlx5	Msx2	[28–30]	*In vitro* binding assays, Y2H
PKA	ATF4	[47]	Calvarial osteoblasts, mouse model
ERK	ATF4	[47]	Calvarial osteoblasts, mouse model
PKA	IGF-I	[48,49]	Bronchial epithelial cells (human), gyrus precursors (mouse)
Wnt pathway	Wnt3a	Website Nusse	Embryonic carcinoma cells
HIF-2α	Ihh	[50]	Mouse reporter assay
δ-EF1	Ihh	[51]	Mouse/chick growth plate, electrophoretic mobility shift assay (EMSA)
ATF4	Ihh	[52,53]	Mouse primary chondrocytes (rib cage), growth plate, TMC23 chondrocytic cells, Ihh luciferase reporter construct
Wnt	Tcf/Lef1	Website Nusse[54]	Human colon cancer, mouse growth plate
Runx2	Tcf7/Lef1	[55,56]	Mouse growth plate, reporter assay
Nkx3.2	Runx2	[57,58]	C3H10T1/2, COS-7 (simian renal cells)
Msx2	Runx2	[59]	Mouse myogenic C2C12 cells, osteoblast-like MC3T3-E1 cells, and the rat osteosarcoma cell line ROS17/2.8
Dlx5	Runx2	[59]	“”
HIF-2α	Runx2	[50,60]	Mouse reporter assay, growth plate
PKA(CREB)	Sox9	[61,62]	ChIP,ATDC5
NFκβ	Sox9	[63,64] vs [65]	Mouse limb bud, C2C12, C3H10T1/2
Sox9	PTHrP	[66]	C3H10T1/2, reporter gene construct
Runx2	Sox9	[67]	Mesenchymal stem cells
Smad3	PTHrP	[68]	Chick sternal chondrocytes
CREB	PTHrP	[69]	Human breast epithelial
Sox9	PPR	[70]	Mouse growth plate
Gli	TGFβ	[71]	Mouse limb bud explant
Smad3	Smad7	Canonical	
Smadcomplex	Smad7	Canonical	
CREB	CCND1	[72,73]	Mouse growth plate
ATF2	CCND1	[72,74]	Mouse growth plate
Gli	CCND1	[75–77]	Human granule cell precursors, drosophila
TGFβ	Dlx5	[78]	C2C12

Msx1,2	Dlx5	DECODE, [28,79]	Mouse calvaria, C3H10T1/2, primary osteoblasts and C2C12 cells (indirect)
Runx2	PI3K	[6,80]	ATDC5, C3H10T1/2 and MC3T3-E1 cells
Runx2	Akt	[6]	“”
TGFβ	Ets1	[81]	mouse mammary NMuMG epithelial cells
TGFβ	IGF-IR	[82,83]	Rat articular chondrocytes
NFκβ	IGF-IR	[84,85]	vascular smooth muscle cells, p65 binding motif
STAT1	IGF-IR	[84]	DECODE
Dlx5	Msx2	[28,86,87]	Apical ectoderm ridge (chick)
Smad3	Msx2	[28,78]	C2C12
BMP-2	Msx2	[88,89]	C2C12, mouse metatarsals
NFκβ	δ-EF1	[90]	MCF-10A cells
Ets1	δ-EF1	[81]	mouse mammary NMuMG epithelial cells
NFκβ	HIF-2α	[50]	Promoter assay

Old references:

Active node	Activated node	Reference	System
Sox9	Sox9	[67,91]	Chick chondrocytes
bFGF	NF-κβ	[92]	Human chondrocytes
BMP	Ihh	[93–97]	Chicken/mice embryo (fetal growth plate)
BMP	Col-X	[96,98]	ATDC5/ chicken chondrocytes (in vivo)
BMP	BMPR	[95]	Canonical
BMP	FGFR1	[95]	Mouse chondrocytes
BMP	STAT1	[95]	Mouse chondrocytes
BMP	GATA4	[99,100]	Mouse cardiomyocytes (P19CL6)
BMP2-4-7	Runx2	[101]	Human C2C12, (mouse premyoblasts), C3H10T1/2 (mouse mesenchymal)n human bone marrow derived cells, RoS 17,2.8 (rat osteosarcoma)
BMP6	Col-X, AP	[101,102]	Chick sternal cephalic chondrocytes
BMP pathway	R-smad	[94,98]	Canonical
BMPR	p38 kinase	[96,103,104]	Mouse embryonic fibroblasts (MEF)
cAMP	PKA	[93]	Canonical
CCND1	Runx2	[105]	Rat chondroprogenitor (RCJ3.1C5.18),

			C3H10T1/2
ERK 1/2	R-smad	[106,107]	Mouse lung, C2C12, MEFs
ERK1/2	Runx2	[108–110]	Human MSC, MC3T3 preosteoblasts (mouse)
FGF	Ras	[111]	Canonical
FGF	ERK	[95,96,112]	Chick embryo, mouse chondrocytes
bFGF	BMP7	[113,114]	Mouse limb bud
FGF18	FGFR3	[93,115–117]	Mice growth plate in vivo
FGF18	Ihh	[94,115]	Mice growth plate in vivo
FGFR1	NF-κβ	[65]	C2C12, T1/2, MC615 (chondrogenic mouse limb)
FGFR3	STAT1	[94,118,119]	Rat chondrosarcoma, murine primary chondrocytes
FGFs	Wnts	[94,120]	Mouse and chick limb buds
GATA4	MEF2C	[121]	Mouse anterior heart field in vivo
Gli	BMP2-7	[122]	HaCaT (Human adult low Calcium Temperature keratinocytes)
Gli	TGFβ1	[122]	“”
Gli	BMP6	[122]	“”
Gli	BMP2	[44,118]	Mouse and human BMP2 promoter
Gli2	PPR	[66,123]	
Gli2	Wnt11, Ptch1	[124,125]	HaCaT, MEFs
Gli2	MMP13	[122]	HaCaT
Gli2	PTHrP	[123,126]	Breast cancer, fetal mouse growth plate
Gli2	BMP4-7	[127]	Mouse and human promoter, osteoblastic Hos and renal COS-7 cells(human osteosarcoma and simian)
Gli2	FGFR1	[128]	Fetal growth plate
Gli2,3	Gli1	[124]	HaCaT, canonical
Gli3	PTHrP	[93,128]	Fetal growth plate
GSK3β	Gli	[129]	Drosophila, canonical
GSK3β	Gli2,3	[75]	Canonical
HDAC4	MEF2C	[36]	Mouse and chick limb bud cells
Ihh	PTHrP	[94,97,130]	Mouse fetal growth plate
Ihh	PPR	[131,132]	Mouse fetal growth plate (indirect)
Ihh	Ptch1, Gli1	[75]	Canonical
Ihh	Wnt3a	[133]	Mouse limb explants,

			primary chondrocytes
Ihh pathway	Gli3	[93,94,98,131,134]	Canonical
MEF2C	Runx2	[16,93,135]	Mouse fetal growth plate
MEF2C	Col-X	[16,36]	Mouse and chick limb bud cells
MEF2C	Smaddlx5	[72]	Mouse branchial arch (enhancer element)
NF-κβ	Smad7	[19,136]	Mv1Lu, COS (simian), and NIH-3T3 (murine fibroblast cell lines)
NF-κβ	BMP2	[44,137,138]	MTC-23 (mouse chondrogenic cell line), mouse fetal growth plate
NF-κβ	Sox9	[65,132]	C2C12, C3H10T1/2, MC615
NF-κβ	MMP13	[92]	Human articular chondrocytes
Nkx3.2	Runx2	[93,94,139,140]	C3H10T1/2, murine (rib) chondrocytes, chick embryo explant
Noggin	BMP	[96,98]	canonical
p38 kinase	Sox9	[103]	MEFs
PKA	Sox9	[91,141,142]	Chicken primary chondrocytes, COS-7, RCS (rat chondrosarcoma)
PKA	Gli2,3	[75]	Canonical
PKA	Col-X	[143]	Human and primary bovine chondrocytes
PKA	HDAC4	[36]	Mouse and chick limb bud cells
PKA	CCND1	[105]	Mouse fetal growth plate, COS, C3H10T1/2, RCJ3.1C5.18
PKA	Runx2	[144]	Chick sternal chondrocytes
PTHrP	cAMP	[93]	canonical
PTHrP	Nkx3.2	[93,140]	Chick embryo explant
R-smad	Dsh	[145]	C57BL/6 and ST2 mice primary bone marrow stromal cells
R-smad	PPR	[146]	C2C12 OB differentiation
Runx2	Col-X	[93,98]	Chick chondrocytes, functional sites in mice and chick promoter
Runx2	Ihh	[93,108,147–149]	Mouse fetal growth plate (+ promoter), MDA-MB-231 breast cancer cell line
FGFR1, FGF2-8	Runx2	[101,150]	MC3T3-E1 mouse calvarial cells,C3H10T1/2, in vivo
Runx2	FGF18	[109]	Mouse fetal growth

			plate
Runx2	MMP13	[147]	Mouse fetal growth plate
Runx2	MEF2C	[147]	Mouse fetal growth plate
Runx2-Smad complex		[108,151]	HeLa & mouse embryo
R-smad	Runx2	[152]	C2C12
Smad1	Col-X	[36]	Mouse and chick limb bud cells
Smad2,3	Col-II	[153]	Human MSCs and SW1353 (human chondrosarcoma)
Smad3	HDAC4	[109]	NIH3T3 (MEFs), ROS17/2.8, primary calvarial mouse osteoblasts
Smad3	Runx2	[101,136]	MC3T3-E1, ROS17/2.8, primary calvarial mouse osteoblasts
Smad3 β-catenin Lef/Tcf complex	Gli2	[154]	HaCaT, HepG2 (human)
Smad4	Smadcomplex	[94,98]	Canonical
Smad7	R-smad	[94,98]	Canonical
Smadcomplex	Runx2	[155]	C2C12
Smadcomplex	Smaddlx5	[156]	C2C12
Smaddlx5	Runx2	[156]	C2C12
Sox9	Col-II	[94,153]	Human MSCs, chondrocytes, SW1353 (canonical)
Sox9	Nkx3.2	[139]	C3H10T1/2, murine (fetal) chondrocytes
Sox9	β-catenin	[12,157]	Mouse limb bud cells, murine primary chondrocytes , L cells (fibroblasts), CHO (chinese hamster ovary), HEK293 (Human embryonic kidney), COS1
Sox9	CCND1	[158]	Mouse fetal growth plate, SW1353 (human)
Sox9	Runx2	[159]	Mouse fetal growth plate, ROS17/2.8, COS7, human CMD1 cartilage (indirect)
STAT1	CKI	[95]	Mouse fetal growth plate
STAT1	Smad7	[136]	U4A cells (human fibrosarcoma), human monocytic leukaemia U937 cells, epidermoid carcinoma A431 cells
STAT1	Ihh	[160]	Mouse fetal growth plate

STAT1	PPR	[114]	Mouse fetal growth plate (indirect)
TGFβ	Sox9	[96,161]	Chick embryos
TGFβ	Ras	[136]	Canonical
TGFβ	Smad3	[109,162]	Canonical
TGFβ	CCND1	[163]	HCS-2/8, human chondrocyte-like cell line
TGFβ1	Smaddlx5	[156]	MC3T3-E1, ROS 17/2.8, and ST2 (mouse bone marrow osteogenic cells), ATDC5, C2C12 cells, C3H10T1/2, 3T3-L1 (adipogenic)
Wnt pathway	β-catenin	[93]	Canonical
Wnt pathway	FGFs	[94]	Chick embryo
Wnt3a	Ras	[164]	Canonical (NIH3T3, L cells)
β-catenin	FGF8	[93]	Mouse *in vivo* (facial), chick limb bud

Reference List

1. Kang JS, Alliston T, Delston R, Derynck R (2005) Repression of Runx2 function by TGF-[beta] through recruitment of class II histone deacetylases by Smad3. EMBO J 24: 2543-2555. 10.1038/sj.emboj.7600729.

2. Zheng L, Baek HJ, Karsenty G, Justice MJ (2007) Filamin B represses chondrocyte hypertrophy in a Runx2/Smad3-dependent manner. J Cell Biol 178: 121-128. jcb.200703113 [pii];10.1083/jcb.200703113 [doi].

3. Guan Y, Chen Q, Yang X, Haines P, Pei M, Terek R, Wei X, Zhao T, Wei L (2012) Subcellular relocation of histone deacetylase 4 regulates growth plate chondrocyte differentiation through Ca2+/calmodulin-dependent kinase IV. American Journal of Physiology - Cell Physiology 303: C33-C40.

4. Yang L, Lawson KA, Teteak CJ, Zou J, Hacquebord J, Patterson D, Ghatan AC, Mei Q, Zielinska-Kwiatkowska A, Bain SD, Fernandes RJ, Chansky HA (2013) ESET histone methyltransferase is essential to hypertrophic differentiation of growth plate chondrocytes and formation of epiphyseal plates. Developmental Biology 380: 99-110.

5. Gaur T, Lengner CJ, Hovhannisyan H, Bhat RA, Bodine PVN, Komm BS, Javed A, van Wijnen AJ, Stein JL, Stein GS, Lian JB (2005) Canonical WNT Signaling Promotes Osteogenesis by Directly Stimulating Runx2 Gene Expression. J Biol Chem 280: 33132-33140.

6. Fujita T, Fukuyama R, Enomoto H, Komori T (2004) Dexamethasone inhibits insulin-induced chondrogenesis of ATDC5 cells by preventing PI3K-Akt signaling and DNA binding of Runx2. J Cell Biochem 93: 374-383. 10.1002/jcb.20192.

7. Fujita T, Azuma Y, Fukuyama R, Hattori Y, Yoshida C, Koida M, Ogita K, Komori T (2004) Runx2 induces osteoblast and chondrocyte differentiation and enhances their migration by coupling with PI3K-Akt signaling. J Cell Biol 166: 85-95.

8. Shirakabe K, Terasawa K, Miyama K, Shibuya H, Nishida E (2001) Regulation of the activity of the transcription factor Runx2 by two homeobox proteins, Msx2 and Dlx5. Genes Cells 6: 851-856.

9. Katoh M, Katoh M (2009) Transcriptional regulation of WNT2B based on the balance of Hedgehog, Notch, BMP and WNT signals. Int J Oncol 34: 1411-1415.

10. Furumatsu T, Ozaki T, Asahara H (2009) Smad3 activates the Sox9-dependent transcription on chromatin. The International Journal of Biochemistry & Cell Biology 41: 1198-1204. doi: DOI: 10.1016/j.biocel.2008.10.032.

11. Furumatsu T, Tsuda M, Taniguchi N, Tajima Y, Asahara H (2005) Smad3 Induces Chondrogenesis through the Activation of SOX9 via CREB-binding Protein/p300 Recruitment. J Biol Chem 280: 8343-8350.

12. Topol L, Chen W, Song H, Day TF, Yang Y (2009) Sox9 Inhibits Wnt Signaling by Promoting β-Catenin Phosphorylation in the Nucleus. Journal of Biological Chemistry 284: 3323-3333.

13. Dy P, Wang W, Bhattaram P, Wang Q, Wang L, Ballock Rá, Lefebvre V (2012) Sox9 Directs Hypertrophic Maturation and Blocks Osteoblast Differentiation of Growth Plate Chondrocytes. Dev Cell 22: 597-609.

14. Jin EJ, Lee SY, Choi YA, Jung JC, Bang OS, Kang SS (2006) BMP-2-enhanced chondrogenesis involves p38 MAPK-mediated down-regulation of Wnt-7a pathway. Mol Cells 22: 353-359.

15. Ciarmatori S, Kiepe D, Haarmann A, Huegel U, Tönshoff B (2007) Signaling mechanisms leading to regulation of proliferation and differentiation of the mesenchymal chondrogenic cell line RCJ3.1C5.18 in response to IGF-I. Journal of Molecular Endocrinology 38: 493-508.

16. McKinsey TA, Zhang CL, Olson EN (2002) MEF2: a calcium-dependent regulator of cell division, differentiation and death. Trends in Biochemical Sciences 27: 40-47. doi: 10.1016/S0968-0004(01)02031-X.

17. Liu D, Kang JS, Derynck R (2004) TGF-[beta]-activated Smad3 represses MEF2-dependent transcription in myogenic differentiation. EMBO J 23: 1557-1566. 10.1038/sj.emboj.7600179.

18. Himpe E, Kooijman R (2009) Insulin-like growth factor-I receptor signal transduction and the Janus Kinase/Signal Transducer and Activator of Transcription (JAK-STAT) pathway. BioFactors 35: 76-81. 10.1002/biof.20.

19. Massagué J, Seoane J, Wotton D (2005) Smad transcription factors. Genes & Development 19: 2783-2810.

20. Kretzschmar M, Doody J, Timokhina I, Massagué J (1999) A mechanism of repression of TGFβ/ Smad signaling by oncogenic Ras. Genes & Development 13: 804-816.

21. Matsuura I, Denissova NG, Wang G, He D, Long J, Liu F (2004) Cyclin-dependent kinases regulate the antiproliferative function of Smads. Nature 430: 226-231. 10.1038/nature02650.

22. Sano Y, Harada J, Tashiro S, Gotoh-Mandeville R, Maekawa T, Ishii S (1999) ATF-2 Is a Common Nuclear Target of Smad and TAK1 Pathways in Transforming Growth Factor-β Signaling. Journal of Biological Chemistry 274: 8949-8957.

23. Sugimori K, Matsui K, Motomura H, Tokoro T, Wang J, Higa S, Kimura T, Kitajima I (2005) BMP-2 prevents apoptosis of the N1511 chondrocytic cell line through PI3K/Akt-mediated NF-κβ activation. J Bone Miner Metab 23: 411-419.

24. Cernotta N, Clocchiatti A, Florean C, Brancolini C (2011) Ubiquitin-dependent degradation of HDAC4, a new regulator of random cell motility. Molecular Biology of the Cell 22: 278-289.

25. Blume-Jensen P, Hunter T (2001) Oncogenic kinase signalling. Nature 411: 355-365. 10.1038/35077225.

26. Diehl JA, Cheng M, Roussel MF, Sherr CJ (1998) Glycogen synthase kinase-3β regulates cyclin D1 proteolysis and subcellular localization. Genes & Development 12: 3499-3511.

27. Paroni G, Cernotta N, Dello Russo C, Gallinari P, Pallaoro M, Foti C, Talamo F, Orsatti L, Steinkühler C, Brancolini C (2008) PP2A Regulates HDAC4 Nuclear Import. Molecular Biology of the Cell 19: 655-667.

28. Ryoo HM, Lee MH, Kim YJ (2006) Critical molecular switches involved in BMP-2-induced osteogenic differentiation of mesenchymal cells. Gene 366: 51-57. doi: DOI: 10.1016/j.gene.2005.10.011.

29. Hassan MQ, Javed A, Morasso MI, Karlin J, Montecino M, van Wijnen AJ, Stein GS, Stein JL, Lian JB (2004) Dlx3 Transcriptional Regulation of Osteoblast Differentiation: Temporal Recruitment of Msx2, Dlx3, and Dlx5 Homeodomain Proteins to Chromatin of the Osteocalcin Gene. Molecular and Cellular Biology 24: 9248-9261.

30. Zhang H, Hu G, Wang H, Sciavolino P, Iler N, Shen MM, Abate-Shen C (1997) Heterodimerization of Msx and Dlx homeoproteins results in functional antagonism. Molecular and Cellular Biology 17: 2920-2932.

31. Ulsamer A, Ortuño M, Ruiz S, Susperregui ARG, Osses N, Rosa JL, Ventura F (2008) BMP-2 Induces Osterix Expression through Up-regulation of Dlx5 and Its Phosphorylation by p38. Journal of Biological Chemistry 283: 3816-3826.

32. Li TF, Gao L, Sheu TJ, Sampson ER, Flick LM, Konttinen YT, Chen D, Schwarz EM, Zuscik MJ, Jonason JH, O'Keefe RJ (2010) Aberrant hypertrophy in Smad3-deficient murine chondrocytes is rescued by restoring transforming growth factor β-activated kinase

1/activating transcription factor 2 signaling: A potential clinical implication for osteoarthritis. Arthritis & Rheumatism 62: 2359-2369.

33. Ding Q, Xia W, Liu JC, Yang JY, Lee DF, Xia J, Bartholomeusz G, Li Y, Pan Y, Li Z, Bargou RC, Qin J, Lai CC, Tsai FJ, Tsai CH, Hung MC (2005) Erk Associates with and Primes GSK-3β for Its Inactivation Resulting in Upregulation of β-Catenin. Molecular Cell 19: 159-170.

34. Doble BW, Woodgett JR (2003) GSK-3: tricks of the trade for a multi-tasking kinase. J Cell Sci 116: 1175-1186.

35. Fang X, Yu SX, Lu Y, Bast RC, Woodgett JR, Mills GB (2000) Phosphorylation and inactivation of glycogen synthase kinase 3 by protein kinase A. Proceedings of the National Academy of Sciences 97: 11960-11965.

36. Kozhemyakina E, Cohen T, Yao TP, Lassar AB (2009) Parathyroid hormone-related peptide represses chondrocyte hypertrophy through a protein phosphatase 2A/histone deacetylase 4/MEF2 pathway. Mol Cell Biol 29: 5751-5762.

37. Liao Y, Hung MC (2010) Physiological regulation of Akt activity and stability. Am J Transl Res 2: 19-42.

38. Ugi S, Imamura T, Maegawa H, Egawa K, Yoshizaki T, Shi K, Obata T, Ebina Y, Kashiwagi A, Olefsky JM (2004) Protein Phosphatase 2A Negatively Regulates Insulin's Metabolic Signaling Pathway by Inhibiting Akt (Protein Kinase B) Activity in 3T3-L1 Adipocytes. Molecular and Cellular Biology 24: 8778-8789.

39. Iwamoto M, Yagami K, Lu Valle P, Olsen BR, Petropoulos CJ, Ewert DL, Pacifici M (1993) Expression and role of c-myc in chondrocytes undergoing endochondral ossification. Journal of Biological Chemistry 268: 9645-9652.

40. Rodriguez-Viciana P, Warne PH, Vanhaesebroeck B, Waterfield MD, Downward J (1996) Activation of phosphoinositide 3-kinase by interaction with Ras and by point mutation. EMBO J 15: 2442-2451.

41. Starkman BG, Cravero JD, Delcarlo M, Loeser RF (2005) IGF-I stimulation of proteoglycan synthesis by chondrocytes requires activation of the PI 3-kinase pathway but not ERK MAPK. Biochem J 389: 723-729.

42. Wang L, Shao YY, Ballock RT (2010) Thyroid hormone-mediated growth and differentiation of growth plate chondrocytes involves IGF-1 modulation of betacatenin signaling. J Bone Miner Res 25: 1138-1146. 10.1002/jbmr.5.

43. Liu H, Holm M, Xie XQ, Wolf-Watz M, Grundstr+Âm T (2004) AML1/Runx1 Recruits Calcineurin to Regulate Granulocyte Macrophage Colony-stimulating Factor by Ets1 Activation. Journal of Biological Chemistry 279: 29398-29408.

44. Sugiura T (1999) Cloning and functional characterization of the 5'-flanking region of the human bone morphogenetic protein-2 gene. Biochem J 338: 433-440.

45. Tootle TL, Rebay I (2005) Post-translational modifications influence transcription factor activity: A view from the ETS superfamily. BioEssays 27: 285-298. 10.1002/bies.20198.

46. Watanabe-Takano H, Takano K, Keduka E, Endo T (2010) M-Ras is activated by bone morphogenetic protein-2 and participates in osteoblastic determination, differentiation, and transdifferentiation. Experimental Cell Research 316: 477-490.

47. Elefteriou F, Benson MD, Sowa H, Starbuck M, Liu X, Ron D, Parada LF, Karsenty G (2006) ATF4 mediation of NF1 functions in osteoblast reveals a nutritional basis for congenital skeletal dysplasiae. Cell Metabolism 4: 441-451.

48. Kawaguchi M, Fujita J, Kokubu F, Ohara G, Huang SK, Matsukura S, Ishii Y, Adachi M, Satoh H, Hizawa N (2010) Induction of insulin-like growth factor-I by interleukin-17F in bronchial epithelial cells. Clinical & Experimental Allergy 40: 1036-1043.

49. Choi YS, Cho HY, Hoyt KR, Naegele JR, Obrietan K (2008) IGF-1 receptor-mediated ERK/MAPK signaling couples status epilepticus to progenitor cell proliferation in the subgranular layer of the dentate gyrus. Glia 56: 791-800.

50. Saito T, Fukai A, Mabuchi A, Ikeda T, Yano F, Ohba S, Nishida N, Akune T, Yoshimura N, Nakagawa T, Nakamura K, Tokunaga K, Chung Ui, Kawaguchi H (2010) Transcriptional regulation of endochondral ossification by HIF-2α during skeletal growth and osteoarthritis development. Nat Med 16: 678-686. 10.1038/nm.2146.

51. Bellon E, Luyten FP, Tylzanowski P (2009) δ-EF1 is a negative regulator of Ihh in the developing growth plate. J Cell Biol 187: 685-699.

52. Wang W, Lian N, Li L, Moss HE, Wang W, Perrien DS, Elefteriou F, Yang X (2009) Atf4 regulates chondrocyte proliferation and differentiation during endochondral ossification by activating Ihh transcription. Development 136: 4143-4153.

53. Wang W, Lian N, Ma Y, Li L, Gallant RC, Elefteriou F, Yang X (2011) Chondrocytic Atf4 regulates osteoblast differentiation and function via Ihh. Development .

54. Chang CF, Serra R (2013) Ift88 regulates Hedgehog signaling, Sfrp5 expression, and β-catenin activity in post-natal growth plate. J Orthop Res 31: 350-356.

55. Komori T (2011) Signaling networks in RUNX2-dependent bone development. J Cell Biochem 112: 750-755. 10.1002/jcb.22994.

56. Mikasa M, Rokutanda S, Komori H, Ito K, Tsang Y, Date Y, Yoshida C, Komori T (2011) Regulation of Tcf7 by Runx2 in chondrocyte maturation and proliferation. J Bone Miner Metab 29: 291-299.

57. Song B, Estrada KD, Lyons KM (2009) Smad signaling in skeletal development and regeneration. Cytokine & Growth Factor Reviews 20: 379-388.

58. Kim DW, Lassar AB (2003) Smad-Dependent Recruitment of a Histone Deacetylase/Sin3A Complex Modulates the Bone Morphogenetic Protein-Dependent Transcriptional Repressor Activity of Nkx3.2. Mol Cell Biol 23: 8704-8717.

59. Lee MH, Kim YJ, Yoon WJ, Kim JI, Kim BG, Hwang YS, Wozney JM, Chi XZ, Bae SC, Choi KY, Cho JY, Choi JY, Ryoo HM (2005) Dlx5 Specifically Regulates Runx2 Type II Expression by Binding to Homeodomain-response Elements in the Runx2 Distal Promoter. J Biol Chem 280: 35579-35587.

60. Yang S, Kim J, Ryu JH, Oh H, Chun CH, Kim BJ, Min BH, Chun JS (2010) Hypoxia-inducible factor-2α is a catabolic regulator of osteoarthritic cartilage destruction. Nat Med 16: 687-693. 10.1038/nm.2153.

61. Zhao L, Li G, Zhou GQ (2009) SOX9 Directly Binds CREB as a Novel Synergism With the PKA Pathway in BMP-2 Induced Osteochondrogenic Differentiation. J Bone Miner Res 24: 826-836. 10.1359/jbmr.081236.

62. Piera-Velazquez S, Hawkins DF, Whitecavage MK, Colter DC, Stokes DG, Jimenez SA (2007) Regulation of the human SOX9 promoter by Sp1 and CREB. Exp Cell Res 313: 1069-1079. doi: DOI: 10.1016/j.yexcr.2007.01.001.

63. Ushita M, Saito T, Ikeda T, Yano F, Higashikawa A, Ogata N, Chung U, Nakamura K, Kawaguchi H (2009) Transcriptional induction of SOX9 by NF-kB family member RelA in chondrogenic cells. Osteoarthritis and Cartilage 17: 1065-1075. doi: 10.1016/j.joca.2009.02.003.

64. Caron MMJ, Emans PJ, Surtel DAM, Cremers A, Voncken JW, Welting TJM, van Rhijn LW (2012) Activation of NF-$\kappa\beta$/p65 Facilitates Early Chondrogenic Differentiation during Endochondral Ossification. PLoS ONE 7: e33467. doi:10.1371/journal.pone.0033467.

65. Sitcheran R, Cogswell PC, Baldwin AS (2003) NF$\kappa\beta$ mediates inhibition of mesenchymal cell differentiation through a posttranscriptional gene silencing mechanism. Genes & Development 17: 2368-2373.

66. Amano K, Hata K, Sugita A, Takigawa Y, Ono K, Wakabayashi M, Kogo M, Nishimura R, Yoneda T (2009) Sox9 Family Members Negatively Regulate Maturation and Calcification of Chondrocytes through Up-Regulation of Parathyroid Hormone-related Protein. Mol Biol Cell 20: 4541-4551.

67. Augello A, De Bari C (2010) The Regulation of Differentiation in Mesenchymal Stem Cells. Hum Gene Ther 21: 1226-1238. doi: 10.1089/hum.2010.173.

68. Pateder DB, Ferguson CM, Ionescu AM, Schwarz EM, Rosier RN, Puzas JE, O'Keefe RJ (2001) PTHrP expression in chick sternal chondrocytes is regulated by TGF-β through Smad-mediated signaling. J Cell Physiol 188: 343-351. 10.1002/jcp.1118.

69. Hamzaoui H, Rizk-Rabin M, Gordon J, Offutt C, Bertherat J, Bouizar Z (2007) PTHrP P3 promoter activity in breast cancer cell lines: Role of Ets1 and CBP (CREB binding protein). Molecular and Cellular Endocrinology 268: 75-84. doi: DOI: 10.1016/j.mce.2007.01.014.

70. Akiyama H, Chaboissier MC, Martin JF, Schedl A, de Crombrugghe B (2002) The transcription factor Sox9 has essential roles in successive steps of the chondrocyte differentiation pathway and is required for expression of Sox5 and Sox6. Genes & Development 16: 2813-2828.

71. Alvarez J, Sohn P, Zeng X, Doetschman T, Robbins DJ, Serra R (2002) TGFβ2 mediates the effects of Hedgehog on hypertrophic differentiation and PTHrP expression. Development 129: 1913-1924.

72. Solomon LA, Bérubé NG, Beier F (2008) Transcriptional regulators of chondrocyte hypertrophy. Birth Defect Res C 84: 123-130. 10.1002/bdrc.20124.

73. Long F, Schipani E, Asahara H, Kronenberg H, Montminy M (2001) The CREB family of activators is required for endochondral bone development. Development 128: 541-550.

74. Beier F, Lee RJ, Taylor AC, Pestell RG, LuValle P (1999) Identification of the cyclin D1 gene as a target of activating transcription factor 2 in chondrocytes. Proceedings of the National Academy of Sciences 96: 1433-1438.

75. Hyman JM, Firestone AJ, Heine VM, Zhao Y, Ocasio CA, Han K, Sun M, Rack PG, Sinha S, Wu JJ, Solow-Cordero DE, Jiang J, Rowitch DH, Chen JK (2009) Small-molecule inhibitors reveal multiple strategies for Hedgehog pathway blockade. Proceedings of the National Academy of Sciences 106: 14132-14137.

76. Oliver TG, Grasfeder LL, Carroll AL, Kaiser C, Gillingham CL, Lin SM, Wickramasinghe R, Scott MP, Wechsler-Reya RJ (2003) Transcriptional profiling of the Sonic hedgehog response: a critical role for N-myc in proliferation of neuronal precursors. Proc Natl Acad Sci U S A 100: 7331-7336. 10.1073/pnas.0832317100 [doi];0832317100 [pii].

77. Duman-Scheel M, Weng L, Xin S, Du W (2002) Hedgehog regulates cell growth and proliferation by inducing Cyclin D and Cyclin E. Nature 417: 299-304. 10.1038/417299a.

78. Lee MH, Kim YJ, Kim HJ, Park HD, Kang AR, Kyung HM, Sung JH, Wozney JM, Kim HJ, Ryoo HM (2003) BMP-2-induced Runx2 Expression Is Mediated by Dlx5, and TGF-β1 Opposes the BMP-2-induced Osteoblast Differentiation by Suppression of Dlx5 Expression. Journal of Biological Chemistry 278: 34387-34394.

79. Ichida F, Nishimura R, Hata K, Matsubara T, Ikeda F, Hisada K, Yatani H, Cao X, Komori T, Yamaguchi A, Yoneda T (2004) Reciprocal Roles of Msx2 in Regulation of Osteoblast and Adipocyte Differentiation. Journal of Biological Chemistry 279: 34015-34022.

80. Beier F, Loeser RF (2010) Biology and pathology of Rho GTPase, PI-3 kinase-Akt, and MAP kinase signaling pathways in chondrocytes. J Cell Biochem 110: 573-580. 10.1002/jcb.22604.

81. Shirakihara T, Saitoh M, Miyazono K (2007) Differential Regulation of Epithelial and Mesenchymal Markers by {delta}EF1 Proteins in Epithelial Mesenchymal Transition Induced by TGF-beta. Molecular Biology of the Cell 18: 3533-3544.

82. Fukumoto T, Sperling JW, Sanyal A, Fitzsimmons JS, Reinholz GG, Conover CA, O'Driscoll SW (2003) Combined effects of insulin-like growth factor-1 and transforming growth factor-[beta]1 on periosteal mesenchymal cells during chondrogenesis in vitro. Osteoarthritis and Cartilage 11: 55-64. doi: DOI: 10.1053/joca.2002.0869.

83. Tsukazaki T, Usa T, Matsumoto T, Enomoto H, Ohtsuru A, Namba H, Iwasaki K, Yamashita S (1994) Effect of Transforming Growth Factor-β on the Insulin-like Growth Factor-I Autocrine/Paracrine Axis in Cultured Rat Articular Chondrocytes. Experimental Cell Research 215: 9-16.

84. Werner H, Maor S (2006) The insulin-like growth factor-I receptor gene: a downstream target for oncogene and tumor suppressor action. Trends in Endocrinology & Metabolism 17: 236-242. doi: DOI: 10.1016/j.tem.2006.06.007.

85. Ma Y, Zhang L, Peng T, Cheng J, Taneja S, Zhang J, Delafontaine P, Du J (2006) Angiotensin II Stimulates Transcription of Insulin-Like Growth Factor I Receptor in Vascular Smooth Muscle Cells: Role of Nuclear Factor-+¦B. Endocrinology 147: 1256-1263.

86. Vieux-Rochas M, Bouhali K, Mantero S, Garaffo G, Provero P, Astigiano S, Barbieri O, Caratozzolo MF, Tullo A, Guerrini L, Lallemand Y, Robert Bt, Levi G, Merlo GR (2013) BMP-Mediated Functional Cooperation between <italic>Dlx5;Dlx6</italic> and <italic>Msx1;Msx2</italic> during Mammalian Limb Development. PLoS ONE 8: e51700. doi:10.1371/journal.pone.0051700.

87. Pan ZZ, Kronenberg MS, Huang DY, Sumoy L, Rogina B, Lichtler AC, Upholt WB (2002) Msx2 Expression in the Apical Ectoderm Ridge Is Regulated by an Msx2 and Dlx5 Binding Site. Biochemical and Biophysical Research Communications 290: 955-961.

88. Lee MH, Javed A, Kim HJ, Shin HI, Gutierrez S, Choi JY, Rosen V, Stein JL, van Wijnen AJ, Stein GS, Lian JB, Ryoo HM (1999) Transient upregulation of CBFA1 in response to bone morphogenetic protein-2 and transforming growth factor β1 in C2C12 myogenic cells coincides with suppression of the myogenic phenotype but is not sufficient for osteoblast differentiation. J Cell Biochem 73: 114-125.

89. Amano K, Ichida F, Sugita A, Hata K, Wada M, Takigawa Y, Nakanishi M, Kogo M, Nishimura R, Yoneda T (2008) Msx2 Stimulates Chondrocyte Maturation by Controlling Ihh Expression. Journal of Biological Chemistry 283: 29513-29521.

90. Chua HL, Bhat-Nakshatri P, Clare SE, Morimiya A, Badve S, Nakshatri H (2006) NF-[kappa]B represses E-cadherin expression and enhances epithelial to mesenchymal transition of mammary epithelial cells: potential involvement of ZEB-1 and ZEB-2. Oncogene 26: 711-724.

91. Kumar D, Lassar AB (2009) The Transcriptional Activity of Sox9 in Chondrocytes Is Regulated by RhoA Signaling and Actin Polymerization. Mol Cell Biol 29: 4262-4273.

92. Muddasani P, Norman JC, Ellman M, van Wijnen AJ, Im HJ (2007) Basic Fibroblast Growth Factor Activates the MAPK and NFκβ Pathways That Converge on Elk-1 to Control Production of Matrix Metalloproteinase-13 by Human Adult Articular Chondrocytes. Journal of Biological Chemistry 282: 31409-31421.

93. Mackie EJ, Ahmed YA, Tatarczuch L, Chen KS, Mirams M (2008) Endochondral ossification: How cartilage is converted into bone in the developing skeleton. Int J Biochem Cell Biol 40: 46-62. doi: 10.1016/j.biocel.2007.06.009.

94. Goldring MB, Tsuchimochi K, Ijiri K (2006) The control of chondrogenesis. J Cell Biochem 97: 33-44. 10.1002/jcb.20652.

95. Yoon BS, Pogue R, Ovchinnikov DA, Yoshii I, Mishina Y, Behringer RR, Lyons KM (2006) BMPs regulate multiple aspects of growth-plate chondrogenesis through opposing actions on FGF pathways. Development 133: 4667-4678.

96. Yoon BS, Lyons KM (2004) Multiple functions of BMPs in chondrogenesis. J Cell Biochem 93: 93-103. 10.1002/jcb.20211.

97. Minina E, Wenzel HM, Kreschel C, Karp S, Gaffield W, McMahon AP, Vortkamp A (2001) BMP and Ihh/PTHrP signaling interact to coordinate chondrocyte proliferation and differentiation. Development 128: 4523-4534.

98. Adams SL, Cohen AJ, Lassová L (2007) Integration of signaling pathways regulating chondrocyte differentiation during endochondral bone formation. J Cell Physiol 213: 635-641. 10.1002/jcp.21262.

99. Monzen K, Shiojima I, Hiroi Y, Kudoh S, Oka T, Takimoto E, Hayashi D, Hosoda T, Habara-Ohkubo A, Nakaoka T, Fujita T, Yazaki Y, Komuro I (1999) Bone morphogenetic proteins induce cardiomyocyte differentiation through the mitogen-activated protein kinase kinase kinase TAK1 and cardiac transcription factors Csx/Nkx-2.5 and GATA-4. Mol Cell Biol 19: 7096-7105.

100. Peterkin T, Gibson A, Patient R (2007) Redundancy and evolution of GATA factor requirements in development of the myocardium. Developmental Biology 311: 623-635. doi: 10.1016/j.ydbio.2007.08.018.

101. Shum L, Nuckolls G (2002) The life cycle of chondrocytes in the developing skeleton. Arthritis Res 4: 94-106. 10.1186/ar396.

102. Grimsrud CD, Romano PR, D'souza M, Puzas JE, Reynolds PR, Rosier RN, O'Keefe RJ (1999) BMP-6 Is an Autocrine Stimulator of Chondrocyte Differentiation. J Bone Miner Res 14: 475-482. 10.1359/jbmr.1999.14.4.475.

103. Pan Q, Yu Y, Chen Q, Li C, Wu H, Wan Y, Ma J, Sun F (2008) Sox9, a key transcription factor of bone morphogenetic protein-2-induced chondrogenesis, is activated through BMP pathway and a CCAAT box in the proximal promoter. J Cell Physiol 217: 228-241. 10.1002/jcp.21496.

104. Stanton LA, Underhill TM, Beier F (2003) MAP kinases in chondrocyte differentiation. Developmental Biology 263: 165-175. doi: 10.1016/S0012-1606(03)00321-X.

105. Zhang M, Xie R, Hou W, Wang B, Shen R, Wang X, Wang Q, Zhu T, Jonason JH, Chen D (2009) PTHrP prevents chondrocyte premature hypertrophy by inducing cyclin-D1-dependent Runx2 and Runx3 phosphorylation, ubiquitylation and proteasomal degradation. J Cell Sci 122: 1382-1389.

106. Kretzschmar M, Doody J, Massagu J (1997) Opposing BMP and EGF signalling pathways converge on the TGF-[beta] family mediator Smad1. Nature 389: 618-622. 10.1038/39348.

107. Sapkota G, Alarcón C, Spagnoli FM, Brivanlou AH, Massagué J (2007) Balancing BMP Signaling through Integrated Inputs into the Smad1 Linker. Molecular Cell 25: 441-454. doi: 10.1016/j.molcel.2007.01.006.

108. Cohen J (2009) Perspectives on RUNX genes: An update. American Journal of Medical Genetics Part A 149A: 2629-2646. 10.1002/ajmg.a.33021.

109. Jonason JH, Xiao G, Zhang M, Xing L, Chen D (2009) Post-translational Regulation of Runx2 in Bone and Cartilage. Journal of Dental Research 88: 693-703.

110. Xiao G, Jiang D, Gopalakrishnan R, Franceschi RT (2002) Fibroblast Growth Factor 2 Induction of the Osteocalcin Gene Requires MAPK Activity and Phosphorylation of the Osteoblast Transcription Factor, Cbfa1/Runx2. J Biol Chem 277: 36181-36187.

111. Goldbeter A, Pourquié O (2008) Modeling the segmentation clock as a network of coupled oscillations in the Notch, Wnt and FGF signaling pathways. Journal of Theoretical Biology 252: 574-585. doi: 10.1016/j.jtbi.2008.01.006.

112. Lunn JS, Fishwick KJ, Halley PA, Storey KG (2007) A spatial and temporal map of FGF/Erk1/2 activity and response repertoires in the early chick embryo. Developmental Biology 302: 536-552. doi: 10.1016/j.ydbio.2006.10.014.

113. Minina E, Kreschel C, Naski MC, Ornitz DM, Vortkamp A (2002) Interaction of FGF, Ihh/Pthlh, and BMP Signaling Integrates Chondrocyte Proliferation and Hypertrophic Differentiation. Dev Cell 3: 439-449. doi: 10.1016/S1534-5807(02)00261-7.

114. Kronenberg HM (2003) Developmental regulation of the growth plate. Nature 423: 332-336. 10.1038/nature01657.

115. Liu Z, Xu J, Colvin JS, Ornitz DM (2002) Coordination of chondrogenesis and osteogenesis by fibroblast growth factor 18. Genes & Development 16: 859-869.

116. Horton WA, Degnin CR (2009) FGFs in endochondral skeletal development. Trends in Endocrinology & Metabolism 20: 341-348. doi: 10.1016/j.tem.2009.04.003.

117. Ohbayashi N, Shibayama M, Kurotaki Y, Imanishi M, Fujimori T, Itoh N, Takada S (2002) FGF18 is required for normal cell proliferation and differentiation during osteogenesis and chondrogenesis. Genes & Development 16: 870-879.

118. Ornitz DM, Marie PJ (2002) FGF signaling pathways in endochondral and intramembranous bone development and human genetic disease. Genes & Development 16: 1446-1465.

119. Sahni M, Ambrosetti DC, Mansukhani A, Gertner R, Levy D, Basilico C (1999) FGF signaling inhibits chondrocyte proliferation and regulates bone development through the STAT-1 pathway. Genes & Development 13: 1361-1366.

120. ten Berge D, Brugmann SA, Helms JA, Nusse R (2008) Wnt and FGF signals interact to coordinate growth with cell fate specification during limb development. Development 135: 3247-3257.

121. Dodou E, Verzi MP, Anderson JP, Xu SM, Black BL (2004) Mef2c is a direct transcriptional target of ISL1 and GATA factors in the anterior heart field during mouse embryonic development. Development 131: 3931-3942.

122. Eichberger T, Sander V, Schnidar H, Regl G, Kasper M, Schmid C, Plamberger S, Kaser A, Aberger F, Frischauf AM (2006) Overlapping and distinct transcriptional regulator properties of the GLI1 and GLI2 oncogenes. Genomics 87: 616-632. doi: 10.1016/j.ygeno.2005.12.003.

123. Sterling JA, Oyajobi BO, Grubbs B, Padalecki SS, Munoz SA, Gupta A, Story B, Zhao M, Mundy GR (2006) The Hedgehog Signaling Molecule Gli2 Induces Parathyroid Hormone-

Related Peptide Expression and Osteolysis in Metastatic Human Breast Cancer Cells. Cancer Research 66: 7548-7553.

124. Laner-Plamberger S, Kaser A, Paulischta M, Hauser-Kronberger C, Eichberger T, Frischauf AM (2009) Cooperation between GLI and JUN enhances transcription of JUN and selected GLI target genes. Oncogene 28: 1639-1651.

125. Lipinski RJ, Gipp JJ, Zhang J, Doles JD, Bushman W (2006) Unique and complimentary activities of the Gli transcription factors in Hedgehog signaling. Experimental Cell Research 312: 1925-1938. doi: 10.1016/j.yexcr.2006.02.019.

126. Miao D, Liu H, Plut P, Niu M, Huo R, Goltzman D, Henderson JE (2004) Impaired endochondral bone development and osteopenia in Gli2-deficient mice. Experimental Cell Research 294: 210-222. doi: 10.1016/j.yexcr.2003.10.021.

127. Kawai S, Sugiura T (2001) Characterization of human bone morphogenetic protein (BMP)-4 and -7 gene promoters: activation of BMP promoters by Gli, a sonic hedgehog mediator. Bone 29: 54-61. doi: 10.1016/S8756-3282(01)00470-7.

128. Koziel L, Wuelling M, Schneider S, Vortkamp A (2005) Gli3 acts as a repressor downstream of Ihh in regulating two distinct steps of chondrocyte differentiation. Development 132: 5249-5260.

129. Jia J, Amanai K, Wang G, Tang J, Wang B, Jiang J (2002) Shaggy/GSK3 antagonizes Hedgehog signalling by regulating Cubitus interruptus. Nature 416: 548-552. 10.1038/nature733.

130. van Donkelaar CC, Huiskes R (2007) The PTHrP-Ihh feedback loop in the embryonic growth plate allows PTHrP to control hypertrophy and Ihh to regulate proliferation. Biomech Model Mechanobiol 6: 55-62.

131. Maeda Y, Schipani E, Densmore MJ, Lanske B (2010) Partial rescue of postnatal growth plate abnormalities in Ihh mutants by expression of a constitutively active PTH/PTHrP receptor. Bone 46: 472-478. doi: 10.1016/j.bone.2009.09.009.

132. Kobayashi T, Chung Ui, Schipani E, Starbuck M, Karsenty G, Katagiri T, Goad DL, Lanske B, Kronenberg HM (2002) PTHrP and Indian hedgehog control differentiation of growth plate chondrocytes at multiple steps. Development 129: 2977-2986.

133. Mak KK, Kronenberg HM, Chuang PT, Mackem S, Yang Y (2008) Indian hedgehog signals independently of PTHrP to promote chondrocyte hypertrophy. Development 135: 1947-1956.

134. Wuelling M, Vortkamp A (2010) Transcriptional networks controlling chondrocyte proliferation and differentiation during endochondral ossification. Pediatric Nephrology 25: 625-631.

135. Arnold MA, Kim Y, Czubryt MP, Phan D, McAnally J, Qi X, Shelton JM, Richardson JA, Bassel-Duby R, Olson EN (2007) MEF2C Transcription Factor Controls Chondrocyte Hypertrophy and Bone Development. Dev Cell 12: 377-389. doi: 10.1016/j.devcel.2007.02.004.

136. Derynck R, Zhang YE (2003) Smad-dependent and Smad-independent pathways in TGF-β family signalling. Nature 425: 577-584. 10.1038/nature02006.

137. Feng JQ, Xing L, Zhang JH, Zhao M, Horn D, Chan J, Boyce BF, Harris SE, Mundy GR, Chen D (2003) NFκβ Specifically Activates BMP-2 Gene Expression in Growth Plate Chondrocytes in Vivo and in a Chondrocyte Cell Line in Vitro. Journal of Biological Chemistry 278: 29130-29135.

138. Wu S, Flint JK, Rezvani G, De Luca F (2007) Nuclear Factorκβ p65 Facilitates Longitudinal Bone Growth by Inducing Growth Plate Chondrocyte Proliferation and Differentiation and by Preventing Apoptosis. J Biol Chem 282: 33698-33706.

139. Yamashita S, Andoh M, Ueno-Kudoh H, Sato T, Miyaki S, Asahara H (2009) Sox9 directly promotes Bapx1 gene expression to repress Runx2 in chondrocytes. Exp Cell Res 315: 2231-2240. doi: 10.1016/j.yexcr.2009.03.008.

140. Provot S, Kempf H, Murtaugh LC, Chung Ui, Kim DW, Chyung J, Kronenberg HM, Lassar AB (2006) Nkx3.2/Bapx1 acts as a negative regulator of chondrocyte maturation. Development 133: 651-662.

141. Huang W, Zhou X, Lefebvre V, de CB (2000) Phosphorylation of SOX9 by cyclic AMP-dependent protein kinase A enhances SOX9's ability to transactivate a Col2a1 chondrocyte-specific enhancer. Mol Cell Biol 20: 4149-4158.

142. Kronenberg HM (2006) PTHrP and Skeletal Development. Annals of the New York Academy of Sciences 1068: 1-13. 10.1196/annals.1346.002.

143. Riemer S, Gebhard S, Beier F, Pöschl E, von der Mark K (2002) Role of c-fos in the regulation of type X collagen gene expression by PTH and PTHrP: Localization of a PTH/PTHrP-responsive region in the human COL10A1 enhancer. J Cell Biochem 86: 688-699. 10.1002/jcb.10260.

144. Li TF, Dong Y, Ionescu AM, Rosier RN, Zuscik MJ, Schwarz EM, O'Keefe RJ, Drissi H (2004) Parathyroid hormone-related peptide (PTHrP) inhibits Runx2 expression through the PKA signaling pathway. Experimental Cell Research 299: 128-136. doi: 10.1016/j.yexcr.2004.05.025.

145. Liu Z, Tang Y, Qiu T, Cao X, Clemens TL (2006) A Dishevelled-1/Smad1 Interaction Couples WNT and Bone Morphogenetic Protein Signaling Pathways in Uncommitted Bone Marrow Stromal Cells. Journal of Biological Chemistry 281: 17156-17163.

146. Susperregui ARG, Viñals F, Ho PWM, Gillespie MT, Martin TJ, Ventura F (2008) BMP-2 regulation of PTHrP and osteoclastogenic factors during osteoblast differentiation of C2C12 cells. J Cell Physiol 216: 144-152. 10.1002/jcp.21389.

147. Hecht J, Seitz V, Urban M, Wagner F, Robinson PN, Stiege A, Dieterich C, Kornak U, Wilkening U, Brieske N, Zwingman C, Kidess A, Stricker S, Mundlos S (2007) Detection of novel skeletogenesis target genes by comprehensive analysis of a Runx2-/- mouse model. Gene Expr Patterns 7: 102-112. doi: 10.1016/j.modgep.2006.05.014.

148. Yoshida CA, Yamamoto H, Fujita T, Furuichi T, Ito K, Inoue Ki, Yamana K, Zanma A, Takada K, Ito Y, Komori T (2004) Runx2 and Runx3 are essential for chondrocyte maturation,

and Runx2 regulates limb growth through induction of Indian hedgehog. Genes & Development 18: 952-963.

149. Pratap J, Wixted JJ, Gaur T, Zaidi SK, Dobson J, Gokul KD, Hussain S, van Wijnen AJ, Stein JL, Stein GS, Lian JB (2008) Runx2 Transcriptional Activation of Indian Hedgehog and a Downstream Bone Metastatic Pathway in Breast Cancer Cells. Cancer Research 68: 7795-7802.

150. Zhou YX, Xu X, Chen L, Li C, Brodie SG, Deng CX (2000) A Pro250Arg substitution in mouse Fgfr1 causes increased expression of Cbfa1 and premature fusion of calvarial sutures. Human Molecular Genetics 9: 2001-2008.

151. Javed A, Bae JS, Afzal F, Gutierrez S, Pratap J, Zaidi SK, Lou Y, van Wijnen AJ, Stein JL, Stein GS, Lian JB (2008) Structural Coupling of Smad and Runx2 for Execution of the BMP2 Osteogenic Signal. Journal of Biological Chemistry 283: 8412-8422.

152. Nishimura R, Hata K, Harris SE, Ikeda F, Yoneda T (2002) Core-binding factor α1 (Cbfa1) induces osteoblastic differentiation of C2C12 cells without interactions with Smad1 and Smad5. Bone 31: 303-312. doi: 10.1016/S8756-3282(02)00826-8.

153. Furumatsu T, Tsuda M, Yoshida K, Taniguchi N, Ito T, Hashimoto M, Ito T, Asahara H (2005) Sox9 and p300 Cooperatively Regulate Chromatin-mediated Transcription. Journal of Biological Chemistry 280: 35203-35208.

154. Dennler S, André J, Verrecchia F, Mauviel A (2009) Cloning of the Human GLI2 Promoter. Journal of Biological Chemistry 284: 31523-31531.

155. Stock M, Otto F (2005) Control of RUNX2 isoform expression: The role of promoters and enhancers. J Cell Biochem 95: 506-517. 10.1002/jcb.20471.

156. Li Yl, Xiao Zs (2007) Advances in Runx2 regulation and its isoforms. Medical Hypotheses 68: 169-175. doi: 10.1016/j.mehy.2006.06.006.

157. Hartmann C (2009) Transcriptional networks controlling skeletal development. Current Opinion in Genetics & Development 19: 437-443. doi: 10.1016/j.gde.2009.09.001.

158. Kawakami Y, Rodriguez-León J, Belmonte JCI (2006) The role of TGFβs and Sox9 during limb chondrogenesis. Current Opinion in Cell Biology 18: 723-729. doi: 10.1016/j.ceb.2006.10.007.

159. Zhou G, Zheng Q, Engin F, Munivez E, Chen Y, Sebald E, Krakow D, Lee B (2006) Dominance of SOX9 function over RUNX2 during skeletogenesis. Proc Natl Acad Sci U S A 103: 19004-19009.

160. Sahni M, Raz R, Coffin JD, Levy D, Basilico C (2001) STAT1 mediates the increased apoptosis and reduced chondrocyte proliferation in mice overexpressing FGF2. Development 128: 2119-2129.

161. Chimal-Monroy J, Rodriguez-Leon J, Montero JA, Gañan Y, Macias D, Merino R, Hurle JM (2003) Analysis of the molecular cascade responsible for mesodermal limb chondrogenesis: sox genes and BMP signaling. Developmental Biology 257: 292-301. doi: 10.1016/S0012-1606(03)00066-6.

162. Akiyama H (2008) Control of chondrogenesis by the transcription factor Sox9. Modern Rheumatology 18: 213-219.

163. Li TF, O'Keefe RJ, Chen D (2005) TGF-beta signaling in chondrocytes. Front Biosci 10:681-8.: 681-688.

164. Yun MS, Kim SE, Jeon SH, Lee JS, Choi KY (2005) Both ERK and Wnt/β-catenin pathways are involved in Wnt3a-induced proliferation. Journal of Cell Science 118: 313-322.
