# Supplementary material for: A Qualitative Model of the Differentiation Network in Chondrocyte Maturation: A Holistic View of Chondrocyte Hypertrophy
Source: PLoS One. 2016 Aug 31;11(8):e0162052. doi: 10.1371/journal.pone.0162052 (PMC5007039; doi:10.1371/journal.pone.0162052)
Supplement: S2 File — (PDF) [file pone.0162052.s002.pdf]

Discussion of the influence of the saturation factor

As discussed in the main text, the saturation factor (SF) is intended to approximate the saturation of biological signalling. Low values of SF lead to the requirement that most of the upstream signals must be present to attain maximal activity. Higher values model more saturation meaning that a subset of upstream (activation) signals is sufficient to reach a maximal activity. To choose the value of the SF the models results can be compared to expression profiles in the growth plate (see Figure 1). Specifically we compare activities in the proliferative and hypertrophic zones. *In silico* activity profiles are obtained by imposing the growth factor activities seen in the growth plate. The imposed profiles are given in Table 1.

The overall match between these profiles is scored by a score function. The activity profiles consist of five distinct categories :

– Present : the node is active in both zones
– None-high : the node is exclusively active in the hypertrophic zone
– High-none : the node is exclusively active in the proliferative zone
– Low-high : the node is more active in the hypertrophic zone
– High-low : the node is more active in the proliferative zone

The score function expresses how well these profiles are matched. The score can vary from 21 (none of the profiles are correct) to 0 when all profiles are matched. In principle, each zone can incur a penalty of 0,5. The total score is 21 minus the sum of the incurred penalties. To assign the score, the stable state in proliferation and hypertrophy is compared to the profiles. The qualitative match with the imposed profiles is determined as follows : for the 'present' profile, when a node's activity is less than 5% in any of the stable states (for each of the zones) a penalty of 0,5 (for a total of one per node) is deducted from the fitness function's output. The penalty for the 'low-high' and 'high-low' profiles is determined from the difference of the node's activity in the proliferative and hypertrophic zones. In the 'low-high' profile, the penalty is determined by $1-2(z_{hypertrophic}-z_{proliferative})$, where $z_{hypertrophic}$ and $z_{proliferative}$ is the

Zone :	Proliferative	Hypertrophic
WNT	1/3	1
IHH	1	1
PTHRP	1	0
FGF	1/3	1
TGFβ	1	1/3
BMP	1/3	1

TABLE 1 – **The profiles imposed in the proliferative and hypertrophic zones.** The activity in each zone is given, with 1 representing 100% activity.

activity in the hypertrophic zone and proliferative zone respectively. Furthermore, this penalty cannot exceed 1 or become negative, so the penalty will amount to 0 when the difference in activity exceeds (or equals) 50%, and attain a maximal value of 1 when the activity in the proliferative zone is higher or equal. The reverse logic applies for the 'high-low' profile. The scoring for the profiles 'none-high' and 'high-none' is analogous but an additional penalty of 0,5 is added when the activity of the state corresponding to 'none' exceeds 5%. To ensure the maximum per node is still 1, the score is determined by $0,5-(z_{hypertrophic}-z_{proliferative})$ for 'none-high', where again the penalty becomes 0 when the difference reaches 50%. Additionally, this penalty cannot exceed 0,5.

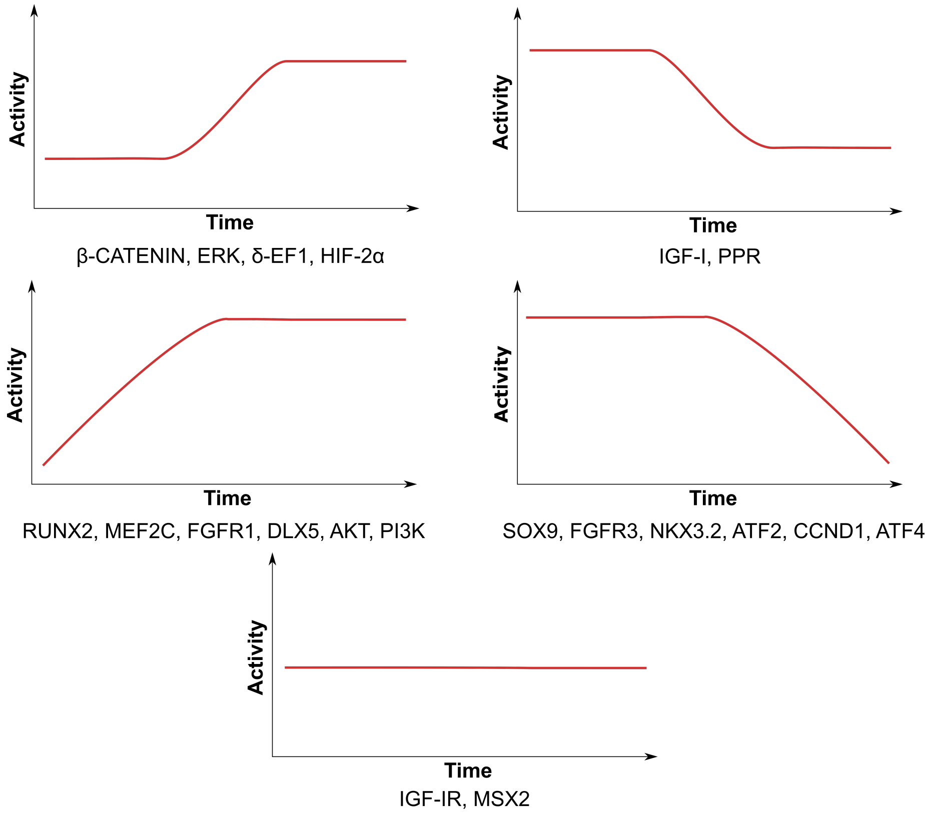

FIGURE 1 – **Graphical representation of the activity profiles.** Each node is listed under the relevant graph. Each graph portrays a category : present / none-high / low-high / high-low / high-none.

The results are shown in Figure 2. As seen in Figure 2, a plateau of optimal fitness is reached roughly between 0,6 and 1. An additional constraint on the solution is that both an attractor with RUNX2 and one with SOX9 must be present.

This additional condition is fullfilled at a value of 0,7. To assess the sensitivity of the results on this optimal plateau, we repeat our analyses for a SF of 0,88. This value was chosen over that of 1 since this is the highest value where a 'None' attractor still exists.

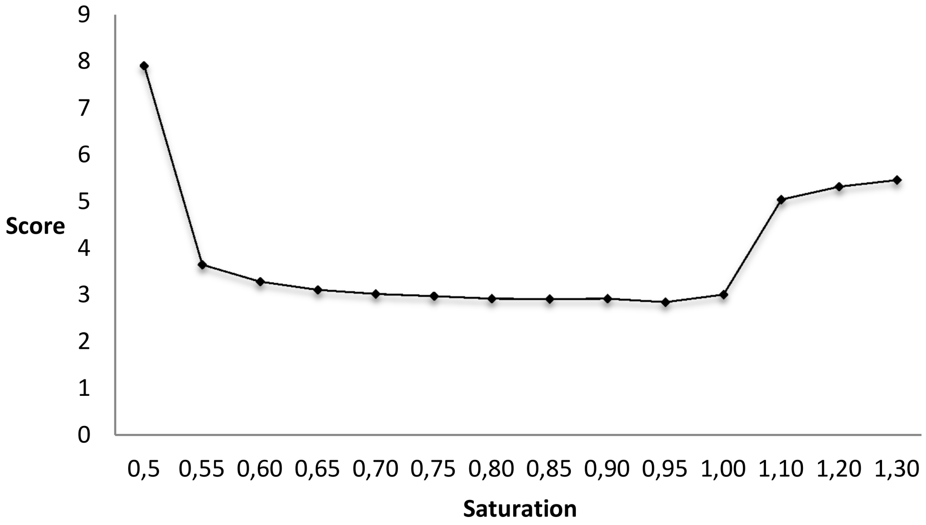

FIGURE 2 – **Saturation (SF) vs the score as assessed by the growth plate profile.** Several values of SF are shown, note that the x-axis is not linear.

The score plateaus at a value of around 3. The value of the score does not reach zero as some errors in the activation profile remain. These errors include the activities of β-CATENIN and δ-EF1, where the difference in activity between the proliferative and hypertrophic zones is not high enough. As such, this error is partly a consequence of the arbitrary cut-off value used by the score function. More definitive errors are found in the profiles of ATF4, CCND1 and MSX2. Finally, MSX2 is not present in the hypertrophic zone, whereas it is detected there in the growth plate (penalty of 0,5). Another error is the presence of CCND1 activity (21 %) in the hypertrophic (RUNX2+) state. For ATF4, the activity in the proliferating zone (SOX9+ state) is erroneous. In fact, the ATF4 activity is almost as high as in the RUNX2 attractor, leading to a penalty of 1. One potential course of action would be to remove the node entirely. We have opted to keep this node as ATF4 activity correlates with Ihh activity in fracture healing, which is a potential application of this model [1].

Though we have used standard formulations of the additive control functions (see supplementary file 3), in many cases a choice between dominant or non-dominant inhibition could be made. Where possible, we have made the choice between dominant and non-dominant inhibition based on the score

function. As discussed above, some discrepancies could not be resolved. In particular, CCND1 activity could not be corrected by either choice. For the case of dominant inhibition, CCND1 was not active in either zone. Therefore, we opted for a non-dominant inhibition as the resulting trend was correct, although its activity was still present in hypertrophy. Similarly, the discrepancies in the profiles of MSX2 and ATF4 could not be resolved in this manner.

Robustness of analyses to value of the saturation factor

As discussed in the main text, results based on robustness in canalisation can significantly differ from the more biologically relevant perturbation analysis. The former is a measure of the stability over the whole of the state space, whereas the latter gives one an idea of the dynamics in the vicinity of the attractors, the area where the cells state will be confined to most of the time if the environment is not overly noisy. This difference in the area to be investigated resulted in the balance between the attractors being significantly altered. Additionally, the balance between attractors is sensitive to changes in saturation factor, as is investigated below. Therefore, our result pertaining to the overall stability of a particular attractor should be interpreted with due caution. Nonetheless, certain trends remain valid irrespective of the value of the saturation factor, including the greater basin of the SOX9 attractor versus the RUNX2 attractor.

To investigate the influence of the saturation factor on the results all simulations were duplicated using a factor of 0,88 instead of 0,7. Firstly, the saturation factor has a large effect on the size of the wild type attractor basin sizes, as seen in Table . The main effect of increasing the saturation factor is a decline in the importance of the None attractor, due to an easier attainability of higher values. This increase benefits both the SOX9 and RUNX2 attractors, though the SOX9 attractor will absorb more if its attractor basin. As such, the SOX9 attractor basin remains dominant, with RUNX2 now forming the 2nd largest attractor basin.

**RUNX2**	11,5 %
**SOX9**	84,7%
**None**	3,8%

TABLE 2 – **Wild type attractor basin size for 0,88 saturation factor.**

For about 6% of outcomes (15 cases) a qualitative difference is observed. Notably this includes the downregulation of FGF and FGFR1, whose effect on RUNX2 adn SOX9 changes. Overall, the results for individual mutants given in Figure 3 are qualitatively similar, indicating that these results do not hinge on a particular value of the saturation factor. Nonetheless, the resolution for certain categories increases. For example, factors that with the 0,7 saturation factor (and the smaller SOX9 basin) completely abolished the SOX9 basin (ATF4, DC) now do not remove it completely. Likewise, nodes that expanded the RUNX2

attractor basin over the entire state space (at least insofar as can be detected by the Monte Carlo analysis), such as WNT, now no longer do so. A more direct comparison is found in Figure 4. Of the effects mentioned in the results section (i.e. the factors that most amplified the SOX9 attractor basin and factors that were required for RUNX2 canalisation) none exhibit a qualitative change.

The differing size of the attractor basins also entails a change in the transition probabilities, the RUNX2 and the None state now have roughly the same stability. The distribution is given in Table . Indeed, transitions to the RUNX2 and mainly to the SOX9 state become more likely at the expense of transitions to the None state. A result is that the transitions to the SOX9 state now become dominant over those to the 'None' attractor. These probabilities hence correlate with the size of the attractor basin. Overall, a higher saturation increases the stability of steady states since the positive feedback loops that keeps them locked in the attractor will be easier to maintain. Figure 5 gives the transitions. The same trend can be seen for the results of the individual nodes. The SOX9 attractor basin is now very dominant and many perturbations are insufficient to leave it. They are given in Figure 6. Most nodes show a less divergent result, in terms of reachable attractors (under different dynamics), since the 'None' attractor can no longer be reached. For instance, inhibitors like MAD7 and δ-EF1 no longer cause a transition from SOX9 to the 'None' attractor. In addition, the upregulation of many nodes no longer suffices to leave the attractor basin, and only the most influential nodes (belonging to the BMP pathway) can effect a transition. In downregulation only the TFGβ and PTHRP pathways are required for the attractor's stability. The usefulness of this analysis declines with the dominance of a particular attractor as unlike the canalisation measure, the perturbation is not permanent. Hence, it becomes increasingly unlikely that an effect will be seen as one state becomes dominant.

Overall, many nodes now flow to the RUNX2 attractor in favour of the None attractor.

**RUNX2**	13,2 %
**SOX9**	71,8%
**None**	15,0%

TABLE 3 – **Steady state of Markov chain for 0,88 saturation factor.**

To conclude, the size of the transition probabilities changes with changing attractor basin size, and due to the dominance of the SOX9 attractor only the nodes with the strongest influence are able to cause a transition. For instance, a knockout of SMAD3 still destabilizes the SOX9 basin, but now flows to the RUNX2 attractor rather than the None attractor. Likewise, the first activation to effect a change from SOX9 to RUNX2 are those of the BMP pathway and this transition is unchanged. However, other stimuli (e.g. from the WNT and FGF pathways) are no longer sufficient. Hence the results of this analysis are dependent the attractor basin size. To an extent, it may be possible to compen-

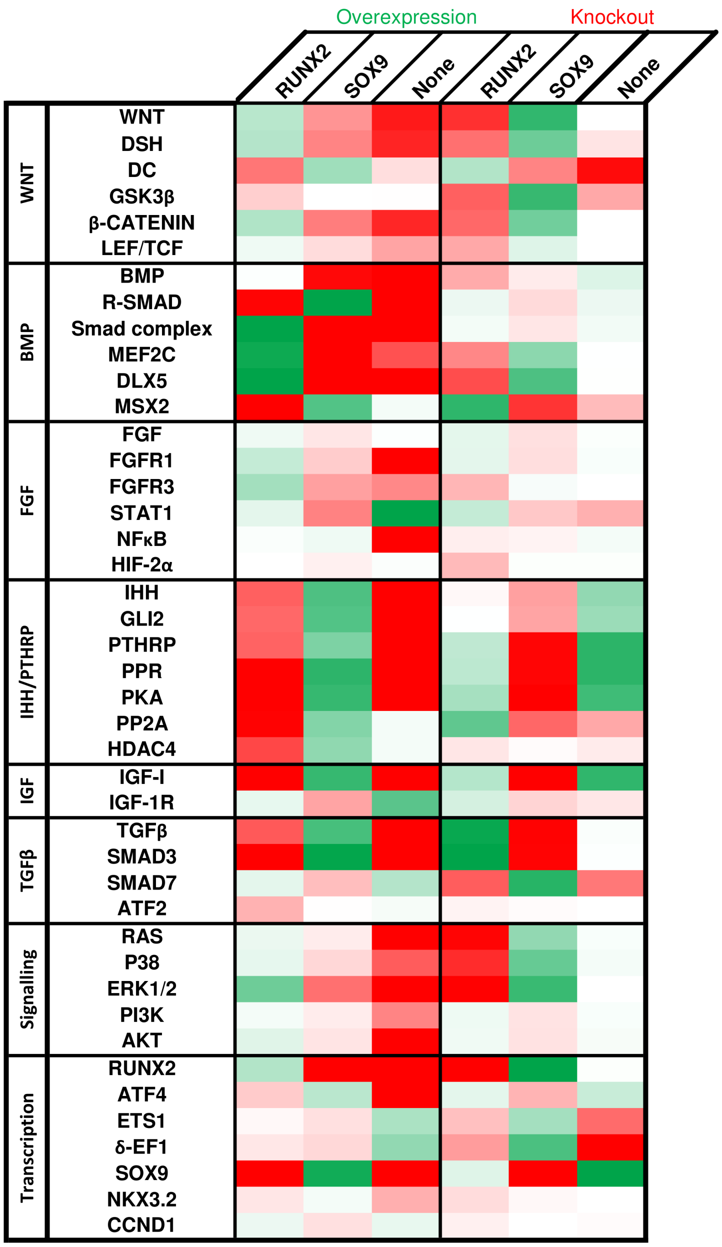

FIGURE 3 – **Attractor basin changes in overexpression and knockout with SF of 0,88.** Changes in the size of attractor basins upon perturbation of the nodes. Both overexpression and knockout are given. Each column is color-coded, up regulation effects are green and down regulation effects are red. Different shades of the colours indicate the intensity of the change. No change is indicated by white.

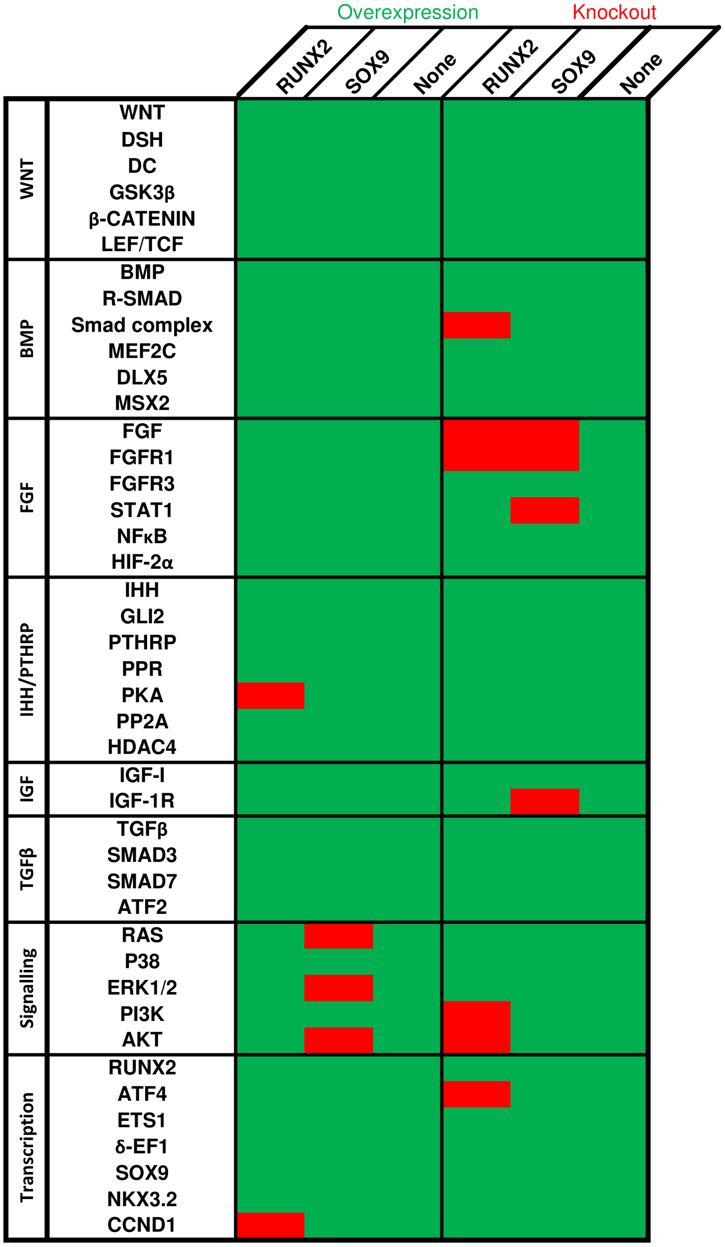

FIGURE 4 – **Comparison results with SF 0,88 and SF 0,7.** The colour is green when the sign of results matches. In case of a mismatch, the corresponding indicator is coloured red. If the change in the original results was less than 2%, this particular outcome is excluded (shown as green).

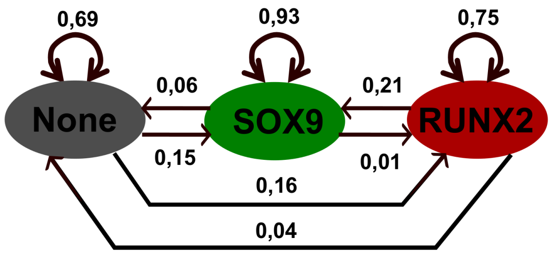

FIGURE 5 – **Markov Chain representation of the network.** Circles represent steady states and the edges transitions between them. The corresponding transition probability is given for each edge.

sate by a larger (upward) perturbation in order to increase sensitivity. However, this would require an adaptation of the framework (as nodes activities are now maximal at 100%). Therefore, the results of the canalisation analysis seem more promising. Indeed, our results indicate that while the size of the attractor basin is less robust to changes in parameters, most (qualitative) results for particular nodes did not share this sensitivity. As can be seen in Figure 3, the effect of perturbations on the attractor basin of individual states is qualitatively unchanged. Hence, the effect of a node on the canalisation of a particular state is seemingly less dependent on parameter changes than it is to its context in the regulatory network.

Comparison between original and adapted topology

As discussed in the main text, some alternative topologies, where WNT signalling is upstream of *Mef2c* (Model 2) or where RUNX2 alone is driving *Mef2c* expression (Model 3), may better capture the results of an *in vitro* experiment. To show that the overall results, presented in the main text, are only affected to a minor degree by this topological change, we repeated the canalisation analysis for Model 3. As seen in Figure 8, the majority of qualitative results are unchanged. Notable exceptions include the effect of Smad complex and BMP on the SOX9 attractor basin, resulting in a better match with *in vitro* measurements.

References

1. Matthews BG, Grcevic D, Wang L, Hagiwara Y, Roguljic H, Joshi P, et al. Analysis of αSMA-Labeled Progenitor Cell Commitment Identifies Notch Signaling as an Important Pathway in Fracture Healing. J Bone Miner Res. 2014 ;29(5) :1283-94. doi : 10.1002/jbmr.2140.

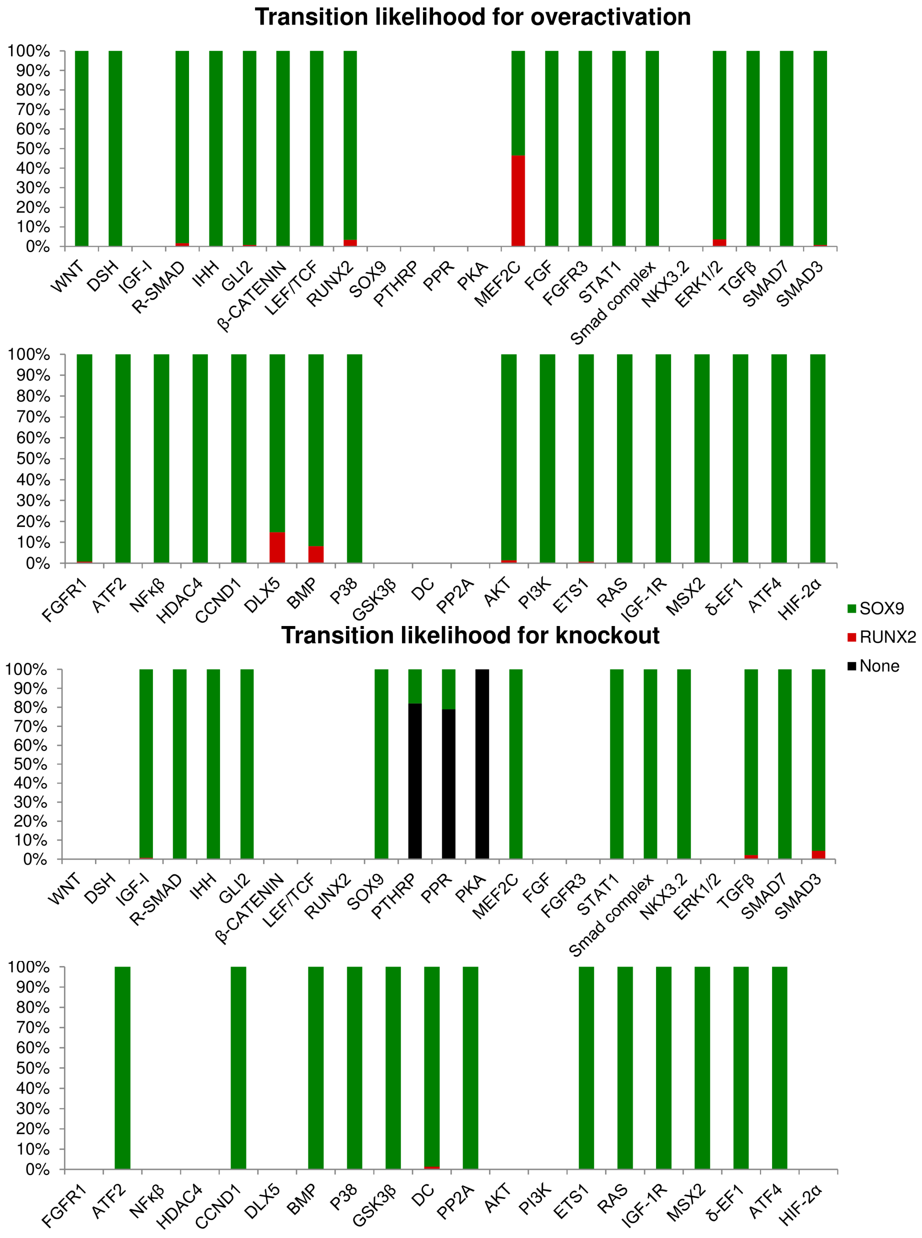

FIGURE 6 – **The effect of perturbations in the SOX9 attractor.** For each node, the average outcomes for three times a hundred perturbations are shown in this figure. The color code indicates the attractor the system settled in after perturbation. Nodes as maximal value are excluded for overactivation and nodes with zero activity are excluded for knockout.

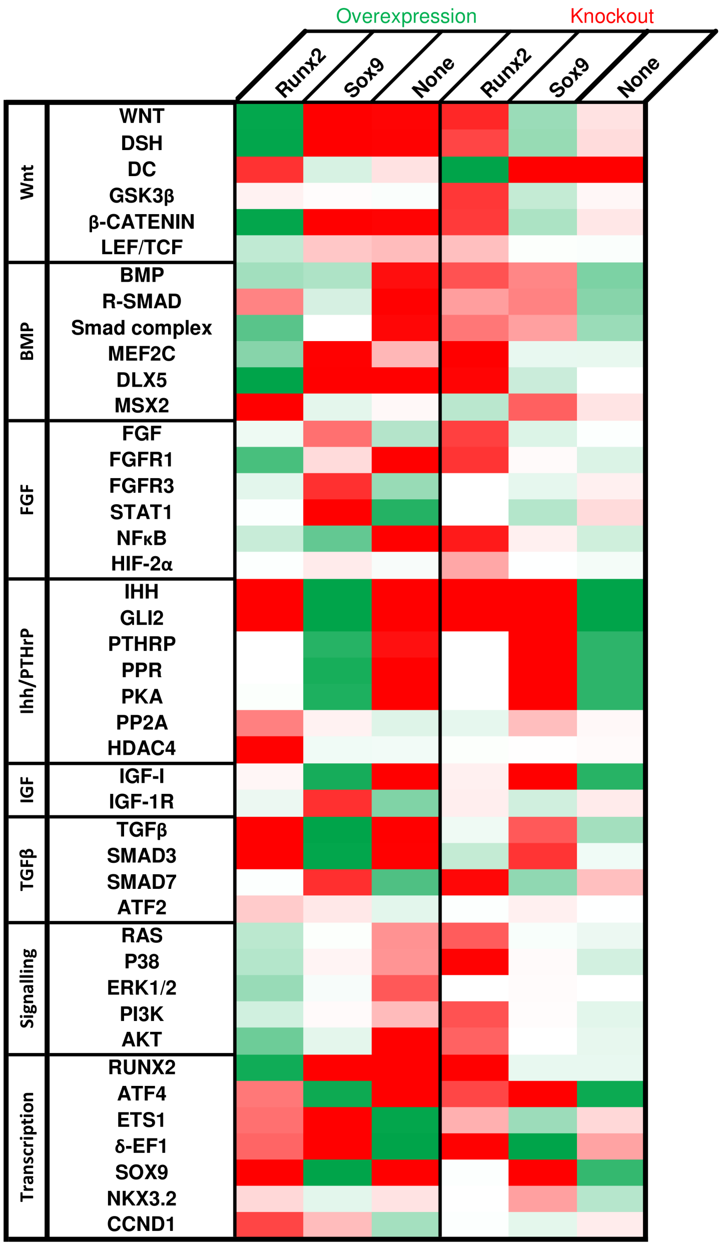

FIGURE 7 – **Attractor basin changes in overexpression and knockout with topology of Model 3.** Changes in the size of attractor basins upon perturbation of the nodes. Both overexpression and knockout are given. Each column is color-coded, up regulation effects are green and down regulation effects are red. Different shades of the colours indicate the intensity of the change. No change is indicated by white.

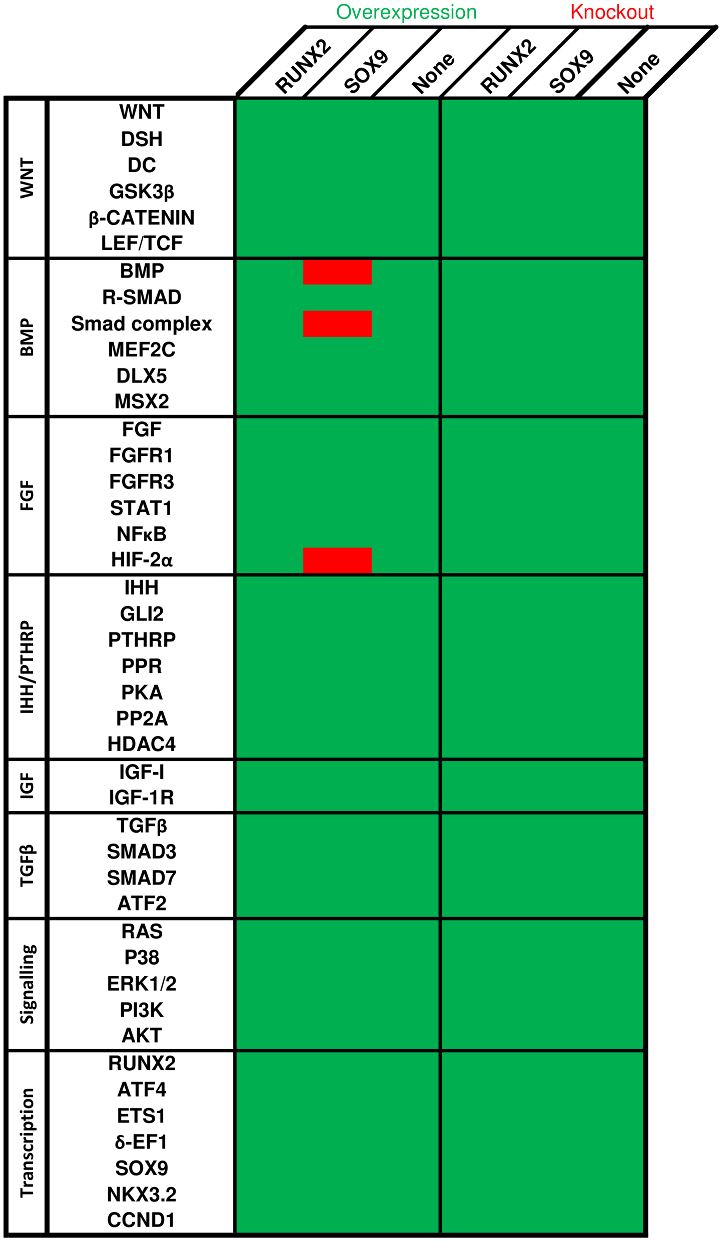

FIGURE 8 – **Comparison results with original and altered topology.** The colour is green when the sign of results matches. In case of a mismatch, the corresponding indicator is coloured red. If the change in the original results was less than 2%, this particular outcome is excluded (shown as green).
