# Supplementary material for: A Qualitative Model of the Differentiation Network in Chondrocyte Maturation: A Holistic View of Chondrocyte Hypertrophy
Source: PLoS One. 2016 Aug 31;11(8):e0162052. doi: 10.1371/journal.pone.0162052 (PMC5007039; doi:10.1371/journal.pone.0162052)
Supplement: S3 File — (PDF) [file pone.0162052.s003.pdf]

Full list of equations of the chondrocyte gene regulatory network.

Note that some factors act in complex (e.g. SMAD3-NKX3.2 inhibition of RUNX2). The regulation of GSK3β is dependent on sequential phosphorylation by ERK and PKA [1,2], which was modelled as a multiplication. For nodes that have majority of inhibitions, these were saturated first (this is the case for SMAD3 and CCND1).

Each gene/node is numbered according to the table below.

Node	Number	Node	Number
WNT	1	SMAD3	26
DSH	2	FGFR1	27
IGF-I	3	ATF2	28
R-SMAD	4	NFκβ	29
IHH	5	HDAC4	30
GLI2	6	CCND1	31
β-CATENIN	7	DLX5	32
LEF/TCF	8	BMP	33
RUNX2	9	P38	34
SOX9	10	GSK3β	35
PTHRP	11	DC	36
PPR	12	PP2A	37
PKA	14	AKT	38
MEF2C	15	PI3K	39
FGF	16	ETS1	40
FGFR3	17	RAS	41
STAT1	18	IGF-IR	42
Smad complex	19	MSX2	43
NKX3.2	21	δ-EF1	44
ERK1/2	22	ATF4	45
TGFβ	23	HIF-2α	46
SMAD7	25		

Each node's activity z is determined by the product of its fast and slow variable ($z = z^f \times z^s$). The standard saturation factor is 2/3. Therefore $s_i = 2 * \frac{\text{saturation factor}}{i}$ where i is the number of excess positive interactions (i > 1).

$$z_1^f(t+1) = 1$$

$$z_1^s(t+1) = z_6(t) + z_7(t)z_8(t) - z_{43}(t)$$

$$z_2^f(t+1) = z_1(t) - 0.5z_4(t)$$

$$z_2^s(t+1) = 1$$

$$z_3^f(t+1) = 1$$

$$z_3^s(t+1) = z_{14}(t)$$

$$z_4^f(t+1) = z_{33}(t) - z_{25}(t)$$

$$z_4^s(t+1) = 1$$

$$z_5^f(t+1) = 1$$

$$z_5^s(t+1) = \left(z_9(t)z_{19}(t) + z_{29}(t) + z_{45}(t) - z_{44}(t)\right)s_2$$

$$z_6^f(t+1) = z_5(t)$$

$$z_6^s(t+1) = 1$$

$$z_7^f(t+1) = 1 - z_{36}(t)$$

$$z_7^s(t+1) = 1$$

$$z_8^f(t+1) = z_7(t) - z_{10}(t)$$

$$z_8^s(t+1) = \left(1 + z_7(t) + z_9(t)\right)s_3$$

$$z_9^f(t+1) = z_{38}(t) + z_{22}(t) + z_{32}(t) + z_{14}(t) - z_{10}(t) - z_{26}(t)z_{30}(t) - z_{31}(t) - z_{43}(t)$$

$$z_9^s(t+1) = \left(z_{46}(t) + z_8(t) + z_9(t) + z_{15}(t) + z_{32}(t) - z_{21}(t)z_{19}(t) - z_{43}(t)\right)s_3$$

$$z_{10}^f(t+1) = z_{14}(t) + z_{26}(t) - z_7(t) - z_9(t)$$

$$z_{10}^s(t+1) = \left(z_{14}(t) + z_{34}(t) + z_{29}(t) + z_{10}(t) + z_{21}(t) - z_{29}(t)\right)s_4$$

$$z_{11}^f(t+1) = 1$$

$$z_{11}^s(t+1) = \left(z_6(t)z_{10}(t) + z_{14}(t) + z_{26}(t)\right)s_3$$

$$z_{12}^f(t+1) = z_{11}(t)$$

$$z_{12}^s(t+1) = \left(z_6(t) + z_{10}(t) + z_{19}(t) - z_{18}(t)\right)s_2$$

$$z_{14}^f(t+1) = \left(z_3(t) + z_{12}(t)\right)s_2$$

$$z_{14}^s(t+1) = 1$$

$$z_{15}^f(t+1) = z_{19}(t) + z_{34}(t) - z_{30}(t) - z_{26}(t)$$

$$z_{15}^s(t+1) = \left(z_9(t) + z_{19}(t)\right)s_2$$

$$z_{16}^f(t+1) = 1$$

$$z_{16}^s(t+1) = \left(z_7(t) + z_9(t)\right)s_2$$

$$z_{17}^{f}(t+1) = z_{16}(t)$$

$$z_{17}^{s}(t+1) = z_{10}(t)$$

$$z_{18}^{f}(t+1) = \big(z_{17}(t) + z_{27}(t) + z_{42}(t) - z_{19}(t)\big)s_2$$

$$z_{18}^{s}(t+1) = 1$$

$$z_{19}^{f}(t+1) = z_{4}(t) - z_{22}(t)$$

$$z_{19}^{s}(t+1) = 1$$

$$z_{21}^{f}(t+1) = 1$$

$$z_{21}^{s}(t+1) = \big(z_{10}(t) + z_{12}(t)\big)s_2$$

$$z_{22}^{f}(t+1) = z_{41}(t) - z_{37}(t)$$

$$z_{22}^{s}(t+1) = 1$$

$$z_{23}^{f}(t+1) = 1$$

$$z_{23}^{s}(t+1) = z_{6}(t)$$

$$z_{25}^{f}(t+1) = 1$$

$$z_{25}^{s}(t+1) = \big(z_{18}(t) + z_{19}(t) + z_{26}(t) + z_{29}(t)\big)s_4$$

$$z_{26}^{f}(t+1) = z_{23}(t) - 0.5s_3\big(z_{25}(t) + z_{22}(t) + z_{31}(t)\big)$$

$$z_{26}^{s}(t+1) = 1$$

$$z_{27}^{f}(t+1) = z_{16}(t) - 0.5z_{19}(t)$$

$$z_{27}^{s}(t+1) = z_{9}(t)$$

$$z_{28}^{f}(t+1) = z_{26}(t)z_{34}(t)$$

$$z_{28}^{s}(t+1) = z_{26}(t)$$

$$z_{29}^{f}(t+1) = \big(z_{27}(t) + z_{38}(t)\big)s_2$$

$$z_{29}^{s}(t+1) = 1$$

$$z_{30}^{f}(t+1) = z_{37}(t) - z_{35}(t)$$

$$z_{30}^{s}(t+1) = 1$$

$$z_{31}^{f}(t+1) = 1 - 0.5s_2\big(z_{35}(t) + z_{18}(t)\big)$$

$$z_{31}^{s}(t+1) = \big(z_{29}(t) + z_{6}(t) + z_{14}(t)\big)s_3$$

$$z_{32}^{f}(t+1) = z_{34}(t) + z_{19}(t) - z_{43}(t)$$

$$z_{32}^{s}(t+1) = z_{15}(t) + z_{19}(t) - z_{26}(t) - z_{43}(t)$$

$$z_{33}^{f}(t+1) = 1$$

$$z_{33}^{s}(t+1) = \left(z_{6}(t) + z_{29}(t)\right)s_{2}$$

$$z_{34}^{f}(t+1) = \left(z_{23}(t) + z_{4}(t)\right)s_{2}$$

$$z_{34}^{s}(t+1) = 1$$

$$z_{35}^{f}(t+1) = 1 - z_{14}(t)z_{22}(t)$$

$$z_{35}^{s}(t+1) = 1$$

$$z_{36}^{f}(t+1) = 1 - z_{2}(t)$$

$$z_{36}^{s}(t+1) = 1$$

$$z_{37}^{f}(t+1) = z_{14}(t)$$

$$z_{37}^{s}(t+1) = 1$$

$$z_{38}^{f}(t+1) = z_{39}(t) - 0.5z_{37}(t)$$

$$z_{38}^{s}(t+1) = z_{9}(t)$$

$$z_{39}^{f}(t+1) = \left(z_{41}(t) + z_{42}(t)\right)s_{2}$$

$$z_{39}^{s}(t+1) = z_{9}(t)$$

$$z_{40}^{f}(t+1) = \left(z_{22}(t) + z_{35}(t)\right)s_{2}$$

$$z_{40}^{s}(t+1) = z_{26}(t)$$

$$z_{41}^{f}(t+1) = \left(z_{1}(t) + z_{33}(t) + z_{17}(t) + z_{27}(t)\right)s_{4}$$

$$z_{41}^{s}(t+1) = 1$$

$$z_{42}^{f}(t+1) = z_{3}(t)$$

$$z_{42}^{s}(t+1) = z_{23}(t) + z_{29}(t) - z_{18}(t)$$

$$z_{43}^{f}(t+1) = 1 - z_{32}(t)$$

$$z_{43}^{s}(t+1) = z_{19}(t) + z_{26}(t) - z_{32}(t)$$

$$z_{44}^{f}(t+1) = 1$$

$$z_{44}^{s}(t+1) = \left(z_{29}(t) + z_{40}(t)\right)s_{2}$$

$z_{45}^{f}(t+1) = \left(z_{14}(t) + z_{22}(t)\right)s_2$

$z_{45}^{s}(t+1) = 1$

$z_{46}^{f}(t+1) = 1$

$z_{46}^{s}(t+1) = z_{29}(t)$

Reference List

1. Fang X, Yu SX, Lu Y, Bast RC, Woodgett JR, Mills GB (2000) Phosphorylation and inactivation of glycogen synthase kinase 3 by protein kinase A. Proceedings of the National Academy of Sciences 97: 11960-11965.

2. Ding Q, Xia W, Liu JC, Yang JY, Lee DF, Xia J, Bartholomeusz G, Li Y, Pan Y, Li Z, Bargou RC, Qin J, Lai CC, Tsai FJ, Tsai CH, Hung MC (2005) Erk Associates with and Primes GSK-3b for Its Inactivation Resulting in Upregulation of b-Catenin. Molecular Cell 19: 159-170.
